# Concentration and avidity of antibodies to different circumsporozoite epitopes correlate with RTS,S/AS01E malaria vaccine efficacy

**DOI:** 10.1038/s41467-019-10195-z

**Published:** 2019-05-15

**Authors:** Carlota Dobaño, Hèctor Sanz, Hermann Sorgho, David Dosoo, Maximilian Mpina, Itziar Ubillos, Ruth Aguilar, Tom Ford, Núria Díez-Padrisa, Nana Aba Williams, Aintzane Ayestaran, Ousmane Traore, Augusto J. Nhabomba, Chenjerai Jairoce, John Waitumbi, Selidji Todagbe Agnandji, Simon Kariuki, Salim Abdulla, John J. Aponte, Benjamin Mordmüller, Kwaku Poku Asante, Seth Owusu-Agyei, Halidou Tinto, Joseph J. Campo, Gemma Moncunill, Ben Gyan, Clarissa Valim, Claudia Daubenberger

**Affiliations:** 1 ISGlobal, Hospital Clínic - Universitat de Barcelona, Rosselló 153, 08036 Barcelona, Catalonia Spain; 2 Centro de Investigação em Saúde de Manhiça (CISM), Rua 12, Cambeve, Vila de Manhiça, CP 1929 Maputo, Mozambique; 3 Unité de Recherche Clinique de Nanoro, Institut de Recherche en Sciences de la Santé, BP 218 Nanoro, Burkina Faso; 4 Kintampo Health Research Centre, P.O. Box 200, Kintampo, Brong-Ahafo Ghana; 5 Ifakara Health Institute, Bagamoyo Research and Training Centre, P.O. Box 74, Bagamoyo, Tanzania; 6 Swiss Tropical and Public Health Institute, Socinstrasse 57, 4002 Basel, Switzerland; 7 University of Basel, Petersplatz 1, 4001 Basel, Switzerland; 8 IAVI - Human Immunology Laboratory, Imperial College, 369 Fulham Road, London, SW10 9NH UK; 9 US Army Medical Research Directorate-Kenya, Walter Reed Army Institute of Research/Kenya Medical Research Institute, Box 54, Kisumu, 40100 Kenya; 10 Centre de Recherches Médicales de Lambaréné (CERMEL), BP 242 Lambaréné, Gabon; 11 Institute of Tropical Medicine and German Center for Infection Research, University of Tübingen, Wilhelmstraße 27, 72074 Tübingen, Germany; 12 Kenya Medical Research Institute/Centre for Global Health, P.O. Box 54840 00200, Kisumu, Siaya, Nairobi, Kenya; 13 Noguchi Memorial Institute for Medical Research, University of Ghana, P.O. Box LG 581, Legon, Ghana; 14 Department of Osteopathic Medical Specialties, Michigan State University, 909 Fee Road, Room B 309 West Fee Hall, East Lansing, MI 48824 USA; 15 Department of Immunology and Infectious Diseases, Harvard T.H. Chen School of Public Health, 675 Huntington Ave., Boston, MA 02115 USA; 16 Department of Global Health, Boston University, 801 Massachusetts Avenue, 3rd Floor, Boston, MA 02118 USA

**Keywords:** Immunology, Infectious diseases, Vaccines, Microbiology

## Abstract

RTS,S/AS01E has been tested in a phase 3 malaria vaccine study with partial efficacy in African children and infants. In a cohort of 1028 subjects from one low (Bagomoyo) and two high (Nanoro, Kintampo) malaria transmission sites, we analysed IgG plasma/serum concentration and avidity to CSP (NANP-repeat and C-terminal domains) after a 3-dose vaccination against time to clinical malaria events during 12-months. Here we report that RTS,S/AS01E induces substantial increases in IgG levels from pre- to post-vaccination (*p* < 0.001), higher in NANP than C-terminus (2855 vs 1297 proportional change between means), and higher concentrations and avidities in children than infants (*p* < 0.001). Baseline CSP IgG levels are elevated in malaria cases than controls (*p* < 0.001). Both, IgG magnitude to NANP (hazard ratio [95% confidence interval] 0.61 [0.48–0.76]) and avidity to C-terminus (0.07 [0.05–0.90]) post-vaccination are significantly associated with vaccine efficacy. IgG avidity to the C-terminus emerges as a significant contributor to RTS,S/AS01E-mediated protection.

## Abstract (web summary)

RTS,S/AS01E has been tested in a phase 3 malaria vaccine trial and has shown partial efficacy in children and infants. Here, the authors analyze IgG concentration and avidity to CSP in ~1000 participants and show that IgG avidity to the C-terminus of CSP is significantly associated with vaccine-mediated protection.

## Introduction

The RTS,S/AS01E is the most advanced malaria vaccine, consistently providing partial protection against clinical malaria in African children^1–11^, although the duration of its moderate vaccine efficacy is limited^3^. Vaccine efficacy against clinical malaria over a 12-month follow-up period estimated in the African phase 3 randomised controlled trial was 55.8% in children (5–17 months old at study start)^1^ and 31.3% in infants (6–12 weeks old at study start)^12^. The vaccine is composed of virus-like particles consisting of the hepatitis B virus surface antigen (HBsAg) and the malarial target antigen, a fragment of the *Plasmodium falciparum* circumsporozoite protein (CSP) spanning the central region containing blocks of four amino acid repeats (NANP) and the C-terminal (C-term) part and formulated with AS01E adjuvant^13^. Previous paediatric phase 1/2^4,5,7,14,15^ and the phase 3^16^ clinical trials in Africa have shown that RTS,S in its various adjuvant formulations induces high IgG antibody titres to CSP NANP repeat that remain above naturally-acquired levels for years^17^. Presently, it is unclear how the vaccine protects against clinical malaria, and why and how the immune mechanism of protection might be affected by age at first immunisation and malaria transmission intensity (MTI)^18^.

Thus far, vaccine immunogenicity measurements have focused mainly on the NANP repeat as the immunodominant B cell epitope^16^. Humoral responses to the subdominant C-term domain have been evaluated in healthy malaria-naïve adults but not in malaria-pre-exposed populations^19^. The C-term domain is structurally conserved^20^ and B cell epitopes in this region have not been characterized. Upon immunisation with *P. falciparum* irradiated sporozoites, monoclonal antibodies against the C-term are rarely induced in malaria naïve volunteers and thus far have not demonstrated to mediate protection^21^. The C-term domain contains a well-known promiscuous CD4^+^ T-cell epitope that is conserved among all parasite isolates^22^.

Although the magnitude of anti-NANP repeat antibodies elicited by RTS,S has been associated with vaccine efficacy in some studies, including the phase 3 trial^16^, others have shown that this response is not associated with protection against clinical malaria^23,24^. The biological function of IgG against the dominant and subdominant CSP epitopes differs, which subsequently could have an impact on protection. Analyses of RTS,S/AS01-induced antibodies in phase 1/2a trials suggested that protected individuals had higher anti-NANP-specific IgG titres but of low opsonisation activity. In contrast, antibodies targeting the C-term in humans were associated with phagocytic activity^19^. Loss of C-term specificity in the overall antibody response to CSP greatly impaired protective efficacy to *P. berghei* in mice^25^. The C-term has been shown to take part in the initial entry of sporozoites into hepatocytes^26^ and therefore, antibodies to this CSP fragment may play a role in protection. There is recent evidence from ongoing studies in our group of an association between post-vaccination HBsAg IgG levels and malaria protection^27^ unreported in previous RTS,S field trials. This might be explained partly by correlation with CSP IgG levels^16^ thus further investigation is required.

In addition to understand epitope fine-specificity of RTS,S/AS01E-induced IgG, it is essential to evaluate quality and quantity of these IgG as a possible indicator of their biological function. Studies conducted in prior phase 2 trials evaluated the avidity of anti-NANP IgG responses in RTS,S vaccinees and found no correlation between avidity post-third dose and vaccine efficacy^28,29^. Changes in NANP IgG avidity and concentration following second and third doses, however, were associated with reduction in clinical malaria risk. A recent phase 2a trial in malaria naïve adults showed that a delayed fractional dose boosting regimen increased antibody avidity and sustained higher protection upon rechallenge^30^. Therefore, these outcomes merit further evaluation as surrogate markers for RTS,S efficacy. Prior phase 2b trials suggested that IgG avidity was higher in children with high malaria exposure compared to those with low malaria exposure one-month post third vaccine dose^28^. The phase 3 trial conducted under a wide range of MTI provides a unique opportunity to address knowledge gaps of RTS,S immunogenicity, immune mechanisms mediating protection, and their determinants.

This study aims to search for antibody correlates of RTS,S/AS01E vaccine-induced protection against malaria by analysing IgG concentrations and avidity to two CSP epitopes (NANP and C-term), and IgG concentrations to HBsAg while considering the effect of age at first vaccination, MTI, and malaria IgG concentrations at baseline. We hypothesise that vaccine efficacy also relies on the contribution of anti-C-term domain binding IgG. Here we show that the magnitude of IgG responses to CSP NANP and C-term and HBsAg, and CSP antibody avidity, are significantly associated with RTS,S/AS01E vaccine efficacy. Our data show that IgG avidity to CSP C-term is important for RTS,S-induced protection against clinical malaria.

## Results

### Study population

RTS,S/AS01E and comparator vaccinees (Supplementary Fig. 1) were similar at baseline with regards to demographic and clinical characteristics, except for sex (Table 1). The majority of subjects (94%) completed the 12-month post-vaccination follow-up time. The median time to drop out of the study was 118 days ranging from 5 to 340 days. Most early terminations were due to migration (33 subjects) or lost to follow-up (26 subjects). A total of 669 malaria clinical events were recorded during the follow-up time (24 in Bagamoyo, 241 in Kintampo and 404 in Nanoro). Of those, 48% were recorded in children and 66% first events occurred within the first six follow-up months. Vaccine efficacy within the first 12 months of follow-up for first (40%; 95% confidence interval [CI] = 29, 49%) and all (27%; 95% CI = 20, 33%) malaria events decreased with time (*p*-Schonfeld test = 0.04) and, as in the phase 3 RTS,S/AS01E trial, varied across age groups (Supplementary Fig. 2). Vaccine efficacy was similar in the three sites: 40% in Nanoro, 43% in Bagamoyo, and 43% in Kintampo (*p* = 0.39, obtained in a Cox Proportional Hazards model testing the interaction between site and vaccination).

**Table 1:** Demographic and clinical characteristics of the whole study population

	RTS,S/AS01E (*n* = 706)	Comparator (*n* = 322)	*p* value
Age at baseline, median (IQR) (weeks)	25 (7, 45)	27 (8, 46)	0.36
Age category, *n* (%), infants (vs children)	290 (41)	119 (37)	0.21
Site, *n* (%)			0.45
Bagamoyo	180 (25)	92 (29)	
Kintampo	221 (31)	90 (28)	
Nanoro	305 (43)	140 (43)	
Sex, *n* (%), male	365 (52)	144 (45)	0.044
Weight, mean ± SD (kg)	6.5 ± 1.9	6.6 ± 1.9	0.56
Weight-for-age *Z*-score, mean ± SD	−0.95 ± 1.21	−0.96 ± 1.23	0.94
Height, median (IQR) (cm)	65 (54, 70)	65 (55, 70)	0.56
Length/height-for-age *Z*-score, mean ± SD	−1.32 ± 1.37	−1.32 ± 1.30	1.00
Number of clinical malaria episodes between pre- (M0) and post- (M3) vaccination, *n* (%)	174 (25)	97 (30)	0.07
1 episode	157 (22)	79 (25)	
2 or more episodes	17 (2)	18 (5)	
Seasonality of transmission at the post-vaccination time point, *n* (%)			0.215
High	390 (55)	162 (50)	
Low or no transmission	316 (45)	160 (50)	
Anaemia^a^, *n* (%)			0.25
No anaemia	142 (20)	71 (22)	
Mild	180 (25)	67 (21)	
Moderate or severe	384 (54)	184 (57)	
Baseline haemoglobin level, mean ± SD (g dL^−1^)	9.74 ± 1.64	9.62 ± 1.74	0.28
Parasitaemia for subjects who had a first clinical malaria episode between M3 + 14 days and M15, Geometric mean ± SD	20,417 ± 18	22,387 ± 17	0.90

*p*-values were estimated using *t*-test where means and SD or geometric means and SD are reported; Wilcoxon Sum Rank test where medians and IQR are reported; Pearson Chi-square or Fisher exact test when proportions are reported

*IQR* interquartile range, *SD* standard deviation, *kg* kilograms, *M* month

^a^Based on the World Health Organization cut off reference values: no anaemia ≥110 g L^−1^, mild anaemia 100–109 g L^−1^, moderate or severe anaemia <99 g L^−1^, http://www.who.int/vmnis/indicators/haemoglobin.pdf?ua=1

### RTS,S/AS01E immunogenicity

**Fig. 1:**
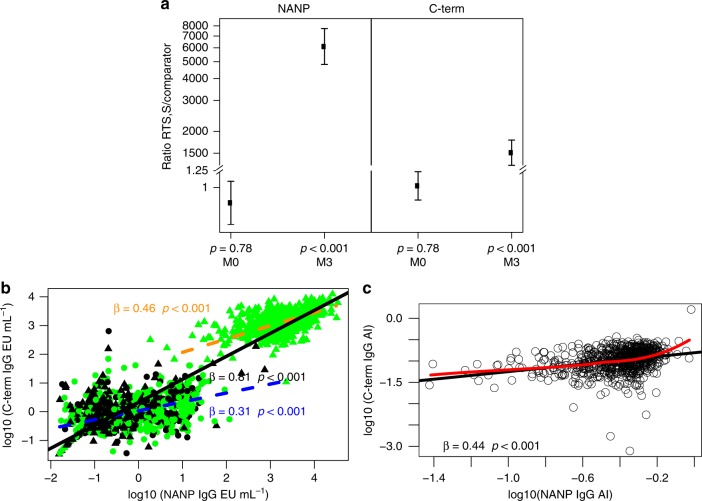
Effect of RTS,S/AS01E vaccination on anti-CSP IgG responses. **a** Ratios of mean concentrations (log_10_[EU mL^−1^]) between RTS,S/AS01E and comparators, before (month [M]0) and after (M3) vaccination, adjusted by site, with adjusted-*p*-values obtained in linear regression coefficients comparing for significance of logarithm of ratios estimated through mixed models. Error bars represent 95% confidence intervals (CI) estimated fitting the mixed model. **b** Correlations between CSP NANP and C-terminus (C-term) IgG concentrations. Regression lines with scatterplots show the association among: (i) all subjects pre-vaccination and post-vaccination in a dashed orange line (*r* = 0.93; 95% CI = 0.93, 0.94); (ii) comparator vaccinees pre-vaccination (black circles) and post-vaccination (black triangles) and RTS,S/AS01E pre-vaccination (green circles) (*r* = 0.48; 95% CI = 0.42, 0.54) in a solid black line; (iii) RTS,S/AS01E post-vaccination (green triangles; *r* = 0.66; 95% CI = 0.62, 0.70) in a solid green colour line. **c** Correlations between CSP NANP and C-term IgG avidity index (AI). Only samples with IgG concentration above the assay lower limit of quantification (LLOQ) (NANP >1.43 EU mL^−1^ and C-term > 2.79 EU mL^−1^) are shown, and those were mostly referring to post-vaccination RTS,S/AS01E vaccinees (*r* = 0.36; 95% CI = 0.29, 0.42). The strength of associations represented by the slope of each regression line is also shown (all slopes with *p* value < 0.05, obtained in linear regression coefficient of the slope, i.e. a *t*-test of the slope )

RTS,S/AS01E vaccination induced significant increase of IgG concentrations (EU mL^−1^) binding to CSP NANP and C-term at M3 compared to M0 (adjusted-*P* < 0.001 comparing logarithm of RTS,S:comparator ratios estimated through mixed models) (Fig. 1a). Percentage of responders was significantly higher in RTS,S-vaccinees (98% NANP, 99% C-term) when compared to comparators (9% NANP, 12% C-term) (*P* < 0.001, Pearson Chi-squared test). The concentration of CSP IgG in RTS,S compared to comparators at post-vaccination (M3) was on average 6000 times higher for NANP and 1600 times higher for C-term (*P* < 0.001 for both ratios). IgG concentration (geometric mean [GM]) and avidity index (AI) at M3 were 1652 and 0.39 for CSP NANP, respectively, and 1241 and 0.10 for C-term. In spite of these differences, the concentrations of IgGs to both CSP antigens were highly correlated (Pearson-*r* = 0.93) (Fig. 1b). Correlations were lower but still moderately high when analysing separately responses pre-vaccination in both groups (Pearson-*r* = 0.48) and post-vaccination in RTS,S/AS01E vaccinees (Pearson-*r* = 0.66), possibly due to the narrower range of concentrations (Fig. 1b). There was a weaker correlation (Pearson-*r* = 0.36) between NANP and C-term AI (Fig. 1c).

### Factors affecting RTS,S/AS01E immunogenicity

**Fig. 2:**
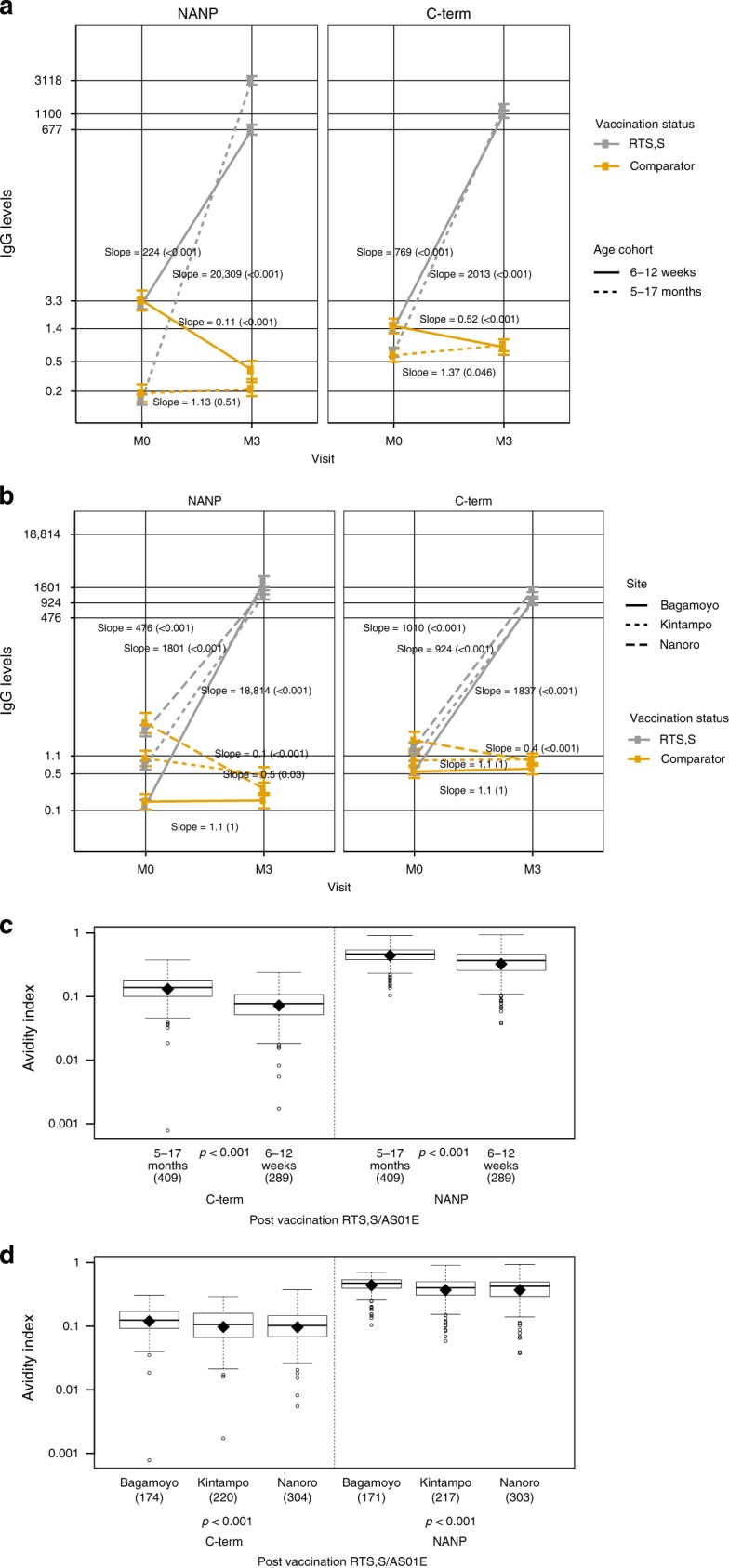
Effect of age cohort and site on RTS,S/AS01E immunogenicity. Comparison of the impact of RTS,S/AS01E vaccination on anti-CSP responses across age cohorts (**a** IgG; **c** AI) and sites (**b** IgG; **d** AI). **a** Estimates of changes over time of infants (aged 6–12 weeks at vaccination) and children (aged 5–17 weeks at vaccination) and statistical significance of those were estimated through multivariate mixed models. Trajectories of NANP of infants and children were significantly different within vaccination group (all *p* values < 0.001). **b** Estimates of changes over time in each study site. Trajectories were statistically significantly different across sites (*p* adjusted for multiple testing for all pairwise comparisons < 0.001). Post-vaccination AI is compared across age cohorts (**c**) and sites (**d**) through *t*-tests and ANOVA, respectively. Boxplots illustrate the medians and the 25th and 75th quartiles, diamonds show the geometric mean, whiskers display the 1.5 interquartile ranges, and dots the outliers. M0: pre-vaccination, M3: post-vaccination

Age at first vaccination and study site significantly affected RTS,S/AS01E immunogenicity (Fig. 2 and Supplementary Figs. 3 and 4; Supplementary Table 2). The magnitude of the change in antibodies from M0 to M3 was significantly greater in children than infants (Fig. 2a): in infants IgG concentrations post-RTS,S vaccination increased from 4750 to 6300 while in children from 6470 to 25,353. Moreover, the changes from pre-vaccination to post-vaccination in children were approximately 10 times larger for NANP and 2.5 times larger for C-term responses than what was observed in the infants cohort. At M0, infants had significantly higher IgG concentrations of CSP NANP and C-term compared to children, most likely attributable to maternally-transferred antibodies (Supplementary Fig. 3c). Higher baseline concentrations of CSP IgG were associated with lower M3 antibody concentrations and lower changes from pre-RTS,S to post-RTS,S vaccination (Supplementary Fig. 5). Percentage sero-positivity did not differ significantly between age groups (Supplementary Table 1).

Volunteers from Nanoro (highest MTI) had higher concentrations of anti-NANP and anti-C-term IgG than those from Kintampo at baseline, and at both sites antibody levels were higher than in Bagamoyo (lowest MTI) (Supplementary Fig. 3d). The antibody increment between M0 and M3 was lesser in sites of high MTI (Fig. 2b; *p* for all pairwise comparison of trajectories between sites < 0.001). Among all other candidate predictors, only weight-for-age *Z*-score (WAZ) had a significant association with immunogenicity (Supplementary Table 2). Sero-positivity in Bagamoyo was lower than in the higher MTI sites and differences were statistically significant for NANP and borderline significant for C-term (Supplementary Table 1).

AI of CSP binding IgG was significantly higher in children than infants (Fig. 2c). Study site had a significant effect on AIs (Fig. 2d), and males had significantly higher AI for C-term IgG than females but no other sex effects were noted (Supplementary Table 2).

### RTS,S/AS01E vaccination induced IgG and malaria protection

**Fig. 3:**
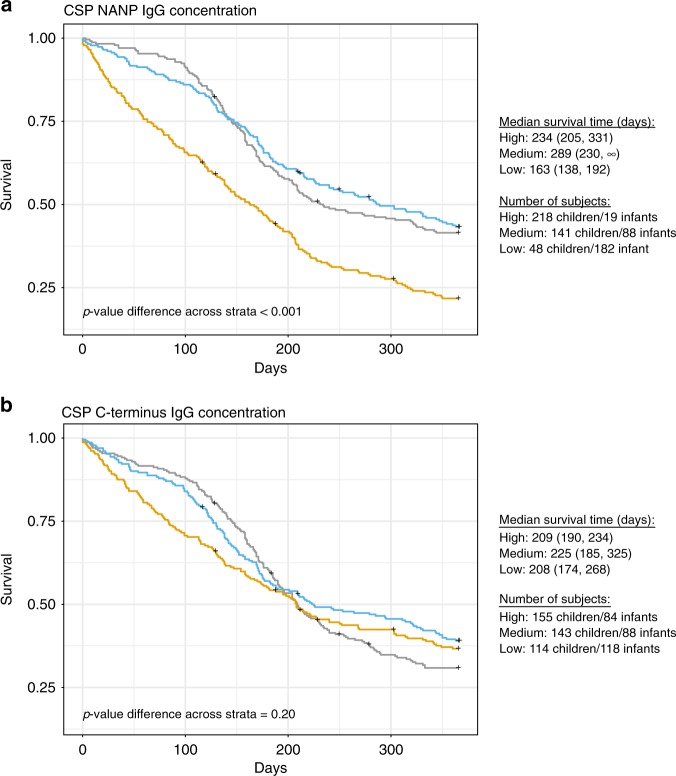
Effect of CSP antibody concentration on protection against the first malaria episode. Kaplan–Meier estimates of time to the first clinical malaria episode during the 12-month follow-up period after the third vaccine dose by the three tertiles (high in grey; medium in blue; low in yellow) of IgG concentrations (EU mL^−1^) to NANP (**a**) and to C-terminus (**b**) at month 3 in RTS,S vaccinees. The median survival time (days), i.e., time after which 50% of the cohort has not yet had an event, is shown for each antibody tertile with the corresponding 95% confidence intervals. *p*-values assessing differences in the distribution of survival time across the three strata in each of the subgroups were estimated through the Log-rank test. The number of subjects in each tertile are also indicated next to the plots, stratified by age group

RTS,S vaccinees exhibiting M3 anti-NANP IgG concentrations at the lower tertile had significantly shorter median time to first clinical malaria event during 12 months of follow-up compared to vaccinees with intermediate or highest anti-NANP IgG concentrations (Fig. 3a). Differences in time to malaria among RTS,S vaccinees across C-term IgG tertile concentrations in the Kaplan–Meier analysis were not statistically significant (Fig. 3b). In contrast, among comparator vaccinees, subjects with high tertiles of IgG NANP concentration at M3 had shorter time to malaria (Supplementary Fig. 6). Kaplan–Meier analysis of sero-positivity could not be done since nearly all RTS,S vaccinees (>98%) had IgG concentrations above the positivity cut-off and data was sparse in the negative category.

Increased continuous concentrations of NANP and C-term protected from first event of clinical malaria after adjusting for age and site, and results were comparable after adjusting for baseline levels (Table 2). Specifically, 10 times increase in anti-NANP IgG concentration was associated with a decrease in malaria hazard by 33%. For C-term, a 11 times increase in IgG concentration was associated with a decrease in malaria hazard by 51%. Although age and site were associated with the hazard of first clinical malaria, they did not modify the hazard ratio (HR) of antibody concentration (*p* of interaction between 0.85 and 0.43). In models including both antigens, NANP IgG concentrations continued to be independently associated with first clinical malaria but not C-term (Table 2).

**Table 2:** IgG antibody concentrations to CSP in relation to risk of clinical malaria

	All subjects at M3				Subjects at M3 with matched M0 sample available			
	NANP	C-term	NANP + C−term	C-term + NANP	NANP	NANP^a^	C-term	C-term^a^
*n* subjects	696	702	694	694	522	522	528	528
Concentration CSP IgG M3	0.69 (0.60, 0.78)	0.77 (0.64, 0.92)	0.61 (0.48, 0.76)	1.21 (0.92, 1.59)	0.71 (0.60, 0.83)	0.73 (0.62, 0.86)	0.86 (0.68, 1.01)	0.84 (0.7, 1.06
Concentration CSP IgG M0	–	–	–	–	–	1.24 (1.03, 1.48)	–	1.30 (1.05, 1.62)
Site								
Bagamoyo	Ref.	Ref.	Ref.	Ref.	Ref.	Ref.	Ref.	Ref.
Kintampo	25.2 (13., 5 47.0)	19.9 (11.2, 35.6)	24.8 (13.3, 46.3)	24.8 (13.3, 46.3)	20.3 (10.7, 38.4)	19.7 (10.4, 37.3)	16.3 (9.1, 29.6)	15.9 (8.8, 28.8)
Nanoro	42.8 (22.9, 79.9)	33.6 (18.8, 60.1)	41.0 (21.9, 76.7)	41.0 (21.9, 76.7)	26.3 (13.1, 52.8)	24.1 (11.9, 48.8)	21.3 (11.1, 41.2)	19.9 (10.27, 38.6)
Age cohort								
5–17 months	Ref.	Ref.	Ref.	Ref.	Ref.	Ref.	Ref.	Ref.
6–12 weeks	0.73 (0.59, 0.91)	0.92 (0.76, 1.13)	0.70 (0.55, 0.88)	0.70 (0.55, 0.88)	0.98 (0.71, 1.36)	0.82 (0.58, 1.17)	1.17 (0.86, 1.61)	1.15 (0.84, 1.57)

Hazard ratios (95% confidence intervals) for the first clinical malaria episode during the 12-month follow-up period after the third vaccine dose in relation to concentrations of IgG antibody against CSP peptides at month (M)3. CSP NANP and C-terminus (C-term) IgG models for RTS,S/AS01E vaccinees are provided including IgG concentration (log_10_ EU mL^−1^), site and age cohort, with or without adjusting for baseline (M0) IgG concentrations. Each column is a single multivariable model for each antibody-antigen predictor for the malaria outcome. Separate models were performed for antibody responses to the two CSP constructs together, and also for the subset of participants who had M0 and M3 matched samples, compared with the full M3 sample analysis

Ref. means the reference variable with which age cohort and site are compared. For example, for the CSP NANP IgG model, Nanoro volunteers had 43× more malaria risk than Bagamoyo, adjusted by age and antibody concentration

^a^Adjusted by baseline (M0) IgG concentrations in the same subjects

**Fig. 4:**
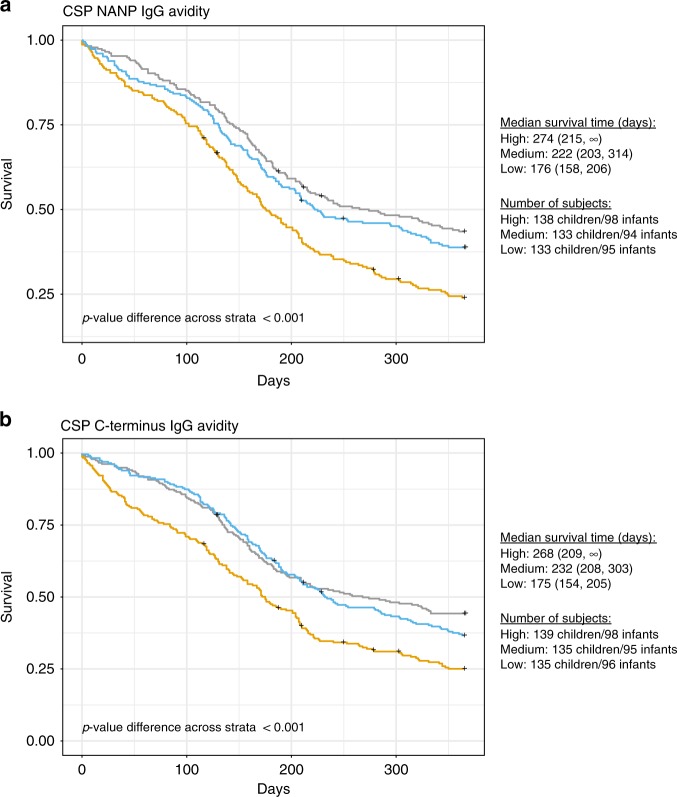
Effect of CSP antibody avidity on protection against the first malaria episode. Kaplan–Meier estimates of time to the first clinical malaria episode during the 12-month follow-up period after the third vaccine dose by the three tertiles (high in grey; medium in blue; low in yellow) of avidity index values at month 3 in RTS,S/AS01E vaccinees. Only plasma/serum samples which IgG concentrations were above the lower limit of quantification (LLOQ) (1.43 EU mL^−1^ and 2.79 EU mL^−1^ in NANP (**a**) and C-terminus [C-term] (**b**), respectively) were included. The median value for NANP was 0.43 and for C-term 0.11 and the number of subjects included was *n* = 691 for NANP and *n* = 698 for C-term. The median survival time (days), i.e., time at which the survivorship function equals 0.5, are shown next to the plots for each strata with the corresponding 95% confidence intervals. *p*-values assessing differences in the distribution of survival time between the two strata were significant and have been estimated through the Log-rank test. The number of subjects in each tertile are also indicated next to the plots, stratified by age group

Survival analysis of AI at M3 showed that RTS,S vaccinees with NANP and C-term values at the lower tertile had significantly shorter median time to first clinical malaria event during 12 months of follow-up than vaccinees with intermediate or highest AI values (Fig. 4). In contrast to IgG concentrations, associations between AI and malaria protection appeared to be consistent for NANP and C-term.

Increased continuous AI of both NANP and C-term IgGs was positively correlated with protection from first clinical malaria event in RTS,S vaccinees (Table 3). Joint analyses of IgG concentration and AI suggest that responses to the two antigens may have distinct behaviours. In those models, C-term AI continued to be independently associated with first clinical malaria but not NANP AI. After adjusting for anti-NANP IgG concentration, the correlation of C-term AI with malaria protection continued to be significant (Table 3) suggesting that the association was independent of the correlation between anti-C-term and anti-NANP responses (Fig. 1b, c). The scatterplots in the Supplementary Fig. 7 suggest that correlation between IgG and AI for C-term is indeed weaker than for NANP. Moreover, for C-term, the association between AI and time to first clinical malaria varied according to IgG concentration. In subjects with low C-term IgG, low AI resulted in shorter median time for clinical malaria (Supplementary Fig. 8). The differences between time to malaria and AI levels decreased in subjects with intermediate C-term IgG concentration. For subjects with high IgG concentration, high C-term AI protected from clinical malaria by delaying the first clinical event. NANP, contrasting with C-term, had comparable effect of AI on clinical malaria across AI levels for subjects with different levels of NANP IgG concentration, suggesting that AI played a lessen role in protection from malaria.

**Table 3:** IgG antibody avidities to CSP in relation to risk of clinical malaria

	Avidity		Avidity + Concentration			
	NANP	C-term	NANP	C-term	C-term	C-term
*n* subjects	691	700	689	700	694	694
Avidity index IgG M3	0.47 (0.23, 0.94)	0.04 (0.01, 0.22)	0.89 (0.43, 1.83)	0.07 (0.05, 0.90)	0.09 (0.02, 0.70)	0.09 (0.03, 0.31)
Concentration NANP IgG M3	–	–	0.54 (0.44, 0.66)	–	0.55 (0.44, 0.70)	0.56 (0.47, 0.67)
Concentration C-term IgG M3	–	–	–	0.70 (0.54, 0.91)	1.00 (0.76, 1.34)	–
Site						
Bagamoyo	Ref.	Ref.	Ref.	Ref.	Ref	Ref.
Kintampo	21.7 (11.7, 40.3)	19.9 (11.2, 35.4)	24.1 (12.9, 44.8)	20.2 (11.4, 35.9)	25.2 (13.6, 46.9)	25.2 (13.6, 47.0)
Nanoro	35.4 (19.1, 65.7)	32.3 (18.2, 55.9)	43.9 (23.5, 82.0)	36.2 (20.3, 64.8)	45.6 (24.3, 85.4)	45.7 (24.4, 85.3)
Age cohort						
5–17 months	Ref.	Ref.	Ref.	Ref.	Ref.	Ref.
6–12 weeks	0.92 (0.75, 1.13)	0.81 (0.65, 1.01)	0.60 (0.47, 0.78)	0.76 (0.61, 0.96)	0.53 (0.41, 0.70)	0.54 (0.41, 0.70)

Hazard ratios (95% confidence intervals) for the first clinical malaria episode during the 12-month follow-up period after the third vaccine dose in relation to IgG antibody avidity index at month (M)3. CSP NANP and C-terminus (C-term) IgG models for RTS,S/AS01E vaccines are provided including avidity index, site and age cohort, with or without adjusting for M3 IgG concentrations of the respective CSP construct and, for C-term, also of the other CSP construct (NANP)

**Fig. 5:**
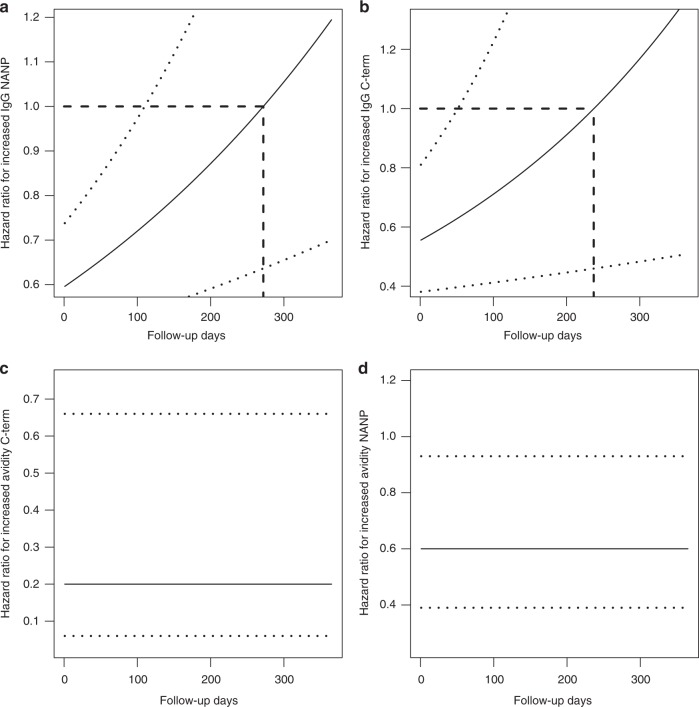
Effect of CSP antibody concentration on protection against multiple malaria episodes. Association between continuous anti-CSP IgG responses and the hazard of recurrent clinical malaria events within a 12-month follow-up period. **a** One unit increase in IgG NANP protects from clinical malaria and is significant (95% confidence interval [CI] excludes 1) in RTS,S/AS01E vaccinees in the beginning of the follow-up period. As times progresses, increased NANP IgG is less protective and becomes not significant (95% CI includes 1) at approximately 120 post-vaccination days. **b** The hazard ratio (HR) of C-terminus (C-term) IgG and recurrent clinical malaria episodes is shown suggesting that increased C-term IgG is significantly protective from clinical malaria until day 90 in RTS,S/AS01E vaccinees. **c** Increased avidity index (AI) to NANP and, in **d** increased AI to C-term, is constantly and significantly associated with a lower risk of clinical malaria in RTS,S/AS01E vaccinees. HRs were estimated in a time-varying proportional hazard model with a HR exponentially varying over time (*p*-value of Schonfeld residual tests for NANP and C-term IgG was <0.001, thus, rejecting a constant HR). Models for IgG concentrations and AIs also included study sites and age cohort with the following HRs: NANP IgG—HR_Kintampo_ = 25 [14,46], HR_Nanoro_ = 31 [17,57], HR_612 weeks, <180 days_ = 2.40 (1.39; 4.14) * [0.99 (0.99; 0.99)]^Days^; HR_612 weeks, >180 days_ = 0.05 (0.02; 0.11) * [1.01 (1.00; 1.01)]^Days^; C-term IgG—HR_Kintampo_ = 20 [11, 35], HR_Nanoro_ = 25 [14, 43], HR_612 weeks, <180 days_ = 3.21 (1.90; 5.41) * [0.99 (0.98; 0.99)]^Days^, HR_612 weeks, >180 days_ = 0.06 (0.03; 0.14) * [1.01 (1.01; 1.01)]^Days^; NANP AI - HR_Kintampo_ = 29 [16, 52], HR_Nanoro_ = 37 [21, 68], HR_612 weeks_ = 1.03 [0.91, 1.18]; C-term AI—HR_Kintampo_ = 23 [14, 40], HR_Nanoro_ = 31 [18, 52], HR_612 weeks_ = 0.98 [0.85, 1.13]

When analysing repeated clinical malaria events, the association between continuous IgG NANP and C-term concentrations varied over time, as expected given prior evidence that vaccine efficacy could wane during the 3–6 months post vaccination (Fig. 5a, b). Increased IgG concentration statistically significantly protected from recurrent clinical malaria for the first 3 months post-vaccination with the HR varying from 0.015 to 0.032. After that, the HR became not statistically significant. From approximately 6.5 months until the end of follow-up at 12 months post-vaccination, the HR was still not statistically significant but ranged from 1 to 1.6, suggesting that lgG to NANP and C-term did not have any effect on the hazard of malaria or marginally increased the risk, perhaps due to unmeasured confounding with exposure to malaria. In contrast, AI in absence of adjustments for IgG concentration was constant and protective of recurrent clinical malaria events for both NANP and C-term (Fig. 5c, d). Conclusions were comparable after adjustments for age and study site, although both factors were associated with recurrent clinical malaria events.

**Fig. 6:**
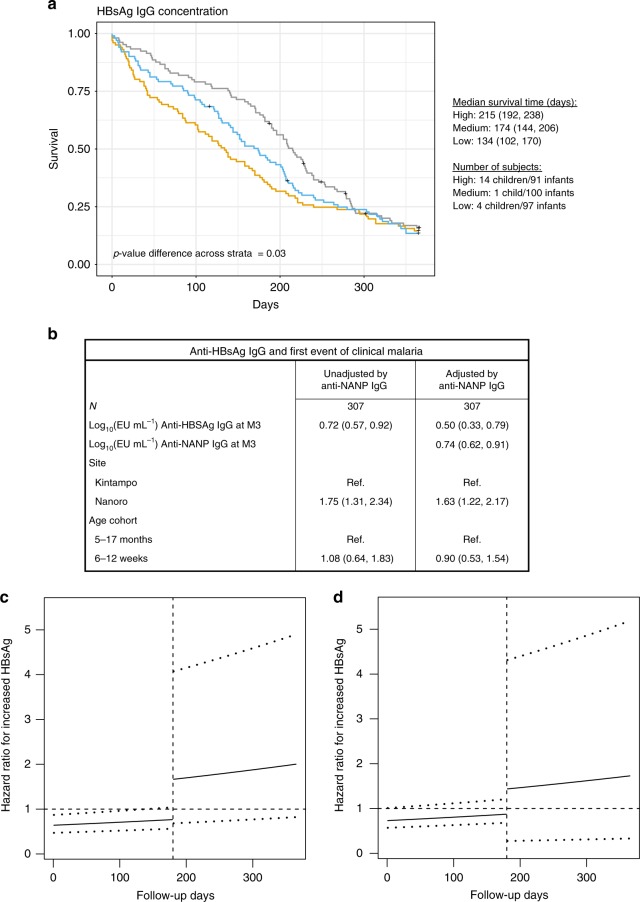
Effect of HBsAg antibody concentration on protection against malaria episodes. Association between categorized anti-HBsAg IgG responses and time to the first clinical malaria episode (**a**), and between continuous anti-HBsAg IgG responses and the hazard of first (**b**) and recurrent (**c**) clinical malaria events within a 12-month follow-up period. **a** Kaplan–Meier curves of time to first clinical malaria for subjects with anti-HBsAg IgG concentration in the upper (grey), intermediate (blue) and lower (yellow) tertiles. Median survival time (and 95% confidence interval [CI]) of subjects in each tertile and sample size stratified by age group are shown, as well as the *p*-value of the log-rank test comparing survival time across tertile-based categories. **b** One unit increase in continuous anti-HBsAg IgG protects from clinical malaria in analysis unadjusted and adjusted for anti-NANP IgG concentration. **c** One unit increase in continuous anti-HBsAg IgG protects from clinical malaria until day 180 and is significantly protective of recurrent events of malaria (95% CI excludes 1) but 180 days after vaccination associations become not statistically significant. Comparable results are shown in **d** when repeating analysis of recurrent malaria events but adjusting for anti-NANP IgG concentration (*p*-value of Schonfeld residual tests for analysis of recurrent events unadjusted for anti-NANP was 0.01 and adjusted for anti-NANP was 0.02, thus, rejecting a constant hazard ratio). All models were adjusted by study site and age cohort

Finally, we found that RTS,S vaccinees (Fig. 6a) (but not comparator vaccinees, Supplementary Fig. 6) in the low tertile of anti-HBsAg IgG concentration had significantly shorter median time to first clinical malaria event than those with higher concentrations. In analysis of continuous anti-HBsAg IgG adjusted by age cohort and site, the protective association of anti-HBsAg IgG concentrations with first (Fig. 6b) and recurrent (Fig. 6c) clinical malaria events was significant for the first six months of follow-up. After adjusting for anti-NANP IgG concentration, the correlation with protection from first (Fig. 6b) and recurrent malaria events (Fig. 6d) of anti-HBsAg IgG concentration continued to be strong suggesting that the association was independent of the correlation between anti-HBsAg IgG and anti-NANP IgG concentrations.

## Discussion

We demonstrate here that anti-CSP IgG concentrations and avidity are correlates of RTS,S induced-protection and that these are affected by age, site and baseline. Our findings confirm data from previous phase 3 analyses showing that RTS,S/AS01E vaccination elicits a potent IgG response to the immunodominant NANP and at higher concentrations in children than infants^3,16^. Importantly, our study was designed and powered to detect immune correlates of protection and to assess the role of age at first vaccination and MTI on immunogenicity. We confirmed that anti-NANP CSP antibodies contribute to protection^16^ against first or recurrent malaria events during 12-months post-immunisation, adjusted by age, site, and also baseline antibodies. This is relevant because correlation between CSP IgG titres and protection against malaria has not always been consistent in previous phase 2b trials, as this association has been dependent on age and/or malaria endpoint (infection vs disease), among other factors^4,5,7,14,17^.

We expanded the analysis of CSP epitope targets by including the C-term domain. We demonstrated that RTS,S/AS01E vaccination induces IgG to this subdominant region and that despite their lower concentrations compared to anti-NANP, these antibodies were also associated with vaccine efficacy.

In addition, we assessed not only the magnitude but also the quality of vaccine-induced antibodies and their relevance to protection. Two field studies of RTS,S evaluated the avidity of anti-NANP CSP IgG in African children and found no correlation between M3 avidity and vaccine efficacy^28,29^. A challenge study in naïve adults showed an association between higher avidity and protection^30^. Here, we showed that malaria-exposed children generated vaccine-induced antibodies with higher AI than infants. This suggests that, in addition to high concentrations, affinity matured antibodies that probably exert better biological functions confer protection against malaria. When multivariable Cox models were adjusted for the other CSP construct and/or the other metric (concentration or AI), the relationship between IgG recognising NANP and malaria protection was different from that of IgG to C-term. Notably, the association between C-term AI and malaria protection remained significant even after adjusting for NANP IgG concentration. Taken together, data indicated that for the NANP region, antibody concentration might be more important in protection than avidity, whereas for the C-term region, the opposite was observed. Indeed, exploratory data analyses pointed to different response patterns in concentrations vs avidities for C-term and NANP. Therefore, the association of one metric with malaria risk was not necessarily confounded by the other metric/antigen.

The magnitude of the HBsAg IgG response was also correlated with time to malaria event, in the first six months after vaccination. This finding could either reflect vaccine take as HBsAg is a component of RTS,S/AS0E, thus related to the correlation of anti-CSP and anti-HBsAg IgG titers (rho ≅ 0.7 in this and other studies)^16,27^, or might also reflect an impact of the HBsAg antigen on the affinity maturation of CSP-specific antibodies resembling the carbohydrate-based conjugate vaccines. As the biological relevance of such association remains unclear, mechanisms and implications for potential interactions between HBsAg and CSP specific responses will be the focus of future studies.

We confirmed that RTS,S/AS01E immunogenicity was higher in the children than in infants. Previous correlates analyses within the phase 3 trial found an association between high tertile M3 NANP IgG titres and vaccine efficacy over the long term follow-up (M30) in infants but not in children^3^. Here, in the analysis of factors affecting the association between antibody concentrations and malaria events, infants had an increased risk compared to children to acquire malaria. The higher antibody concentrations and AI in children compared to infants could partially explain why RTS,S is more efficacious in older ages^1–3^. The nature and kinetics of IgG affinity maturation to dominant (NANP) vs subdominant (C-term) epitopes could be different, and this may also impact the age-related pattern of association between antibodies and malaria protection.

Furthermore, we assessed if the concentration of baseline antibodies to RTS,S antigens affected the outcome of vaccination at the individual level. Presence of CSP antibodies at time of first vaccination due to maternally-transferred antibodies during the first months of life and/or to malaria exposure could impact RTS,S immunogenicity, and this may affect infants and children differentially. Indeed, baseline concentrations of anti-CSP IgG were higher in subjects from Nanoro than those in Kintampo or Bagamoyo. We have shown previously that baseline levels of CSP IgG in infants aged less than 10 months (thus presumably transferred in utero from the mother) correlate positively and significantly with baseline levels of IgG to *P. falciparum* antigens—therefore representing markers of malaria exposure^27^. In children older than 10 months, baseline CSP IgGs would likely be acquired upon infection and increase with age and MTI. A prior analysis of the effect of anti-NANP CSP IgG within the phase 3 trial reported that pre-vaccination titres were associated with lower RTS,S immunogenicity in infants and higher immunogenicity in children^16^. We confirm a significant correlation between higher M0 and lower M3 CSP NANP IgG concentrations and expand this for C-term IgG. These associations were significant when infants and children were analysed together, but they were more prominent for NANP IgGs and for infants. Subjects who developed malaria during the 12-month follow-up period had higher concentrations of IgG CSP at baseline and induced lower concentrations of IgG at M3, thus the increments from M0 to M3 in the non-protected subjects were lower than in protected volunteers. Thus, naturally-acquired CSP antibodies (at M0, and at M3 in comparator vaccinees) were markers of exposure, while RTS,S/AS01E-induced CSP (M3) are markers of protection. Malaria episodes during the vaccination period were also associated with increased malaria risk post-vaccination. As malaria exposure may affect the phenotype and function of immune cells needed for antibody production, less exposed children at M0 appear able to generate higher responses at M3, and may benefit most from RTS,S vaccination. This may be important for the deployment of vaccines in general as prior parasite clearance may increase vaccine take.

Finally, we explored the effect on vaccination and protection of other anthropometric variables not analysed thus far. We found some associations for sex and WAZ, but patterns were not consistent to draw conclusions. The impact of nutrition on vaccine take merits further investigation in larger studies in case an intervention could improve effectiveness.

One limitation of our study design was that some baseline sera were missing, particularly for children from Nanoro, and thus we could not evaluate the baseline effects at the individual level in the whole study population. In addition, only 5–17-months-old children were enrolled at Bagamoyo for the immunology study as per local protocol. Thus, the findings related to site could be confounded by the effect of age. Therefore, further studies are warranted to assess in more depth the effect of maternally-transferred antibodies and exposure to other *P. falciparum* antigens at baseline in relation to M3 peak and long-term maintenance of responses.

In conclusion, we generated data on RTS,S/AS01E immunogenicity including the C-term of CSP and evaluated antibody avidity as a quality index essential for immune effector mechanisms. While our current efforts are devoted to better define the type and subclasses of antibodies elicited by RTS,S vaccination, next steps include the assessment of the functionality of CSP-specific IgGs in regards to protection, and the evaluation of the response kinetics with and without a fourth booster dose at month 20. The elucidation of the mechanisms underlying RTS,S/AS01E-induced partial protection is essential for guiding rational development and deployment of next generation vaccines.

## Methods

### Study design and subjects

This ancillary immunology study (MAL067) was nested in the phase 3 RTS,S/AS01E randomised controlled trial (MAL055, ClinicalTrials.gov NCT00866619) conducted between 2009 and 2014 in sub-Saharan African countries with differing MTIs, including two age cohorts: infants aged 6–12 weeks and children aged 5–17 months at first vaccine dose. Volunteers were vaccinated with either a comparator vaccine or a course of three doses of RTS,S/AS01E given at study months 0, 1 and 2 (M0, M1, and M2). Serum or plasma was collected at baseline (M0), and approximately 30 days after the third vaccine dose (M3). Samples selected were from a subset of 1028 subjects enrolled in 3 out of 7 trial sites participating in the MAL067 study (Kintampo-Ghana, Nanoro-Burkina Faso, Bagamoyo-Tanzania) and had (i) one-year cumulative incidence high enough to yield 80% power to detect moderate HRs, (ii) available M3 samples from infants and children (only children in Bagamoyo as per local protocol), and (iii) varying MTI (Kintampo moderate-high, Nanoro high-seasonal, Bagamoyo low)^16^. These three sites were prioritized because they collectively fulfilled the above criteria due to availability of sufficient numbers and volumes of samples to address the study objectives. The follow-up by passive case detection (PCD) for clinical malaria events (fever ≥ 37.5 °C and parasitaemia of any density) was defined for the 12 months starting 14 days after the third vaccine dose. The authors have complied with all relevant ethical regulations for work with human participants. Written informed consent was obtained from parents or guardians before the start of the work. The study protocol was approved by the following relevant institutional or national Ethics Committees: National Institute for Medical Research, Ifakara Health Institute (IHI IRB), Tanzania; Ethics Committee of the University and State of Basel (EKBB), Switzerland; Kintampo Health Research Centre (KHRC) Institutional Ethics Committee (IEC), and Noguchi Memorial Institute for Medical Research IRB, Ghana; Institutional EC IRSS and *Comite d’Ethique pour la Recherche en Sante*, Burkina Faso; *Comitè Ètic d’Investigació Clínica* (CEIC, Hospital Clínic, UB), Barcelona, Spain; Research Ethics Committee (REC), PATH, USA.

### CSP antigens

The NANP synthetic peptide used for this study was based on 6 copies of the CSP central repeat sequence (H_2_NNANPNANPNANPNANPNANPNANP-C-COOH). The C-term peptide covered 66 amino acids (Ahx-EPSDKHIKEYLNKIQNSLSTEWSPCSVTCGNGIQVRIKPGSANKPKDELDYANDIEKKICKMEKCS-NH_2_). Both antigens were sourced by IAVI-HIL from JPT peptide Technologies (Berlin, Germany). Antigen coating concentration was at 1 μg mL^−1^.

### ELISA assays

IgG titration and avidity assays were performed on the same plate at IAVI-HIL. Each plate included 6 samples run in two sets of duplicates and 2-step 12-fold serial dilutions (1:50, 1:600, 1:7200). Antigen-specific standard curves were implemented using monoclonal antibodies of known concentration (μg mL^−1^) (provided by PATH-MVI) specific to either C-term or NANP. Curves were assayed in duplicate and 9-step 3-fold serial dilutions starting at 1:200. Positive, negative and blank controls were assayed in each plate in duplicate. Negative control sera were obtained from healthy adults living in non-endemic malaria areas (seronegative for CSP). The positivity threshold was defined as the mean + 2 standard deviations (SD) of the negative controls (C-term = 2.95 EU mL^−1^, NANP = 1.78 EU mL^−1^). Positive controls were a pool of serum samples from RTS,S/AS01-vaccinated individuals (provided by GSK). Acceptance limits were established prior to processing samples based on Westgard rules for this positive control.

The indirect ELISA was performed using 1:6000 rabbit anti-human IgG HRP conjugate (DAKO, Cat No P0214), Sureblue TMB Peroxidase substrate (KPL, Cat No 52-00-02) and TMB Stop Solution (KPL, Cat No 50-85-06). For the avidity assay, 1 set of test samples and positive control duplicate wells were incubated for 30 min with a chaotropic agent (1 M Ammonium thiocyanate, Cat No 12960594, Fisher Scientific, UK), whilst the 2nd set of the same samples in duplicate wells on the plate were incubated with buffer alone. IgG concentration was reported in EU mL^−1^ units. The AI was defined as the ratio of interpolated IgG EU mL^−1^ after incubating with chaotropic agent divided by the equivalent value from the ELISA assay without chaotropic agent and multiplied by 100. Standards, negative controls and blanks were also incubated in buffer and were not treated with chaotropic agent. For the analysis of AI, we only included samples which anti-CSP IgG concentrations were above the assay lower limits of quantification (LLOQ) provided by IAVI-HIL (for NANP > 1.43 EU mL^−1^ and for C-term > 2.79 EU mL^−1^).

### Data analysis

For the descriptive analysis of demographic, clinical, and other characteristics of subjects between RTS,S/AS01E and comparator vaccinees, categorical variables were compared through summary statistics, Pearson Chi-square or Fisher tests, and continuous variables through *t*-tests or Wilcoxon rank-sum tests, as appropriate. Graphical and basic exploratory comparisons between vaccination groups were done through boxplots and *t*-tests (Welch corrected). Associations between antibodies to different antigens were sought through linear regression and correlation coefficients.

Mixed linear models investigated the effect of the vaccine on the logarithm_10_-transformed IgG concentrations for each antigen at M3 and change of concentrations from M0 to M3 through tests of the corresponding fixed effects (via combinations of the estimated coefficients), including random intercept and interaction terms. Additionally, in those models, we considered adjustments for age cohorts and sites and compared the effect of vaccination on changes of IgG concentrations from M0 to M3 and on concentrations at M3 across age cohorts and sites through interaction tests.

The impact of age cohort and site on AI focused on antibodies at M3, only in RTS,S vaccinees with anti-CSP IgG concentrations greater than the LLOQ. Analysis was based on correlation, linear regression, and *t*-tests of log_10_ AI.

Assessment of the impact of IgG concentration and AI on protection was primarily based on time to first clinical malaria event following the parent trial main endpoint but, as a secondary endpoint, recurrent malaria events were analysed. First, Kaplan–Meier time-to-event estimates were compared across tertile-based categories of antibody responses within vaccination groups through log-rank tests. In Kaplan–Meier analysis of AI, only samples with IgG concentration above the LLOQ were included. Second, the effect of each M3 continuous IgG response (CSPs and HBsAg concentrations; AI CSPs) on the prevention of clinical malaria was assessed by proportional hazard models that used Anderson and Gill approach when including recurrent events as the outcome^31^. Given prior publications suggesting that vaccine efficacy started declining 3–6 months after vaccination^2,32^, we were concerned that the protection conferred by IgGs could wane and be time-varying, assuming that antibodies mediated the effect of RTS,S/AS01E vaccine efficacy (or were surrogates). Therefore, the assumption of proportional hazards or constant hazard ratio over follow-up time was assessed through tests of Schoenfeld residuals. When the proportional hazards assumption was rejected, the functional representation of the hazard over time that resulted in the best model fitness was chosen. Optimality of model fitness was based on Akaike Information Criterion (AIC). In analysis of time to first event, only IgG C-term concentration would require use of time-varying hazards and, thus, for simplicity of reporting, a constant hazard was forced in. In analyses of recurrent events, an exponential hazard over time attained the lowest AIC and, thus, yielded optimal model fitness. Moreover, the HR between age cohort and recurrent clinical malaria as a function of time categorized at 167–186 days, attained an optimal fit. We chose to adjust models for age categorized at 180 days or 6 months in all models, for simplicity. We reported graphically the time-varying hazard ratio for recurrent events of increased antibody responses.

Concentration of IgG to HBsAg from participants with M3 samples available (162 [52%] from Kintampo and 277 [62%] from Nanoro) was obtained from the MAL055 trial database and used to analyse the association between HBsAg antibody titres and malaria vaccine efficacy in this study. Analyses of the hazard of recurrent clinical malaria episodes attained optimal fitness per AIC with HBsAg as a piecewise exponential function split at around 180 follow-up days, depending on the model. Again, for simplicity and given reports of waning vaccine efficacy at about 180 days, we chose to standardize models in a split at 180 follow-up days.

In all regression models we considered adjustments for candidate confounders: sex, WAZ, height-for-age *Z*-score (HAZ), reported clinical malaria prior to vaccination, and malaria transmission season, defined according to the rainy months at the time of M3 sample collection: high for Bagamoyo March–May and November–December, for Nanoro July–December, and for Kintampo April–October. Linearity of associations with continuous covariates was evaluated through penalized splines in generalized additive models (GAM) and no variable was deemed non-linearly associated. Covariates statistically significant to a 0.05 level or that impacted the coefficient of IgG concentration or AI were retained in final adjusted models. As a result, no covariate other than age and site, were kept in proportional hazards models. All analyses were stratified by vaccination group and, in regression models we considered stratification by age cohorts and site when interaction terms were significant to a 0.05 alpha-level. When analysing immunogenicity and comparing sites, *p*-values were corrected for multiple testing through Benjamini-Hochberg and considered significant if <0.05. Analyses were conducted using R v3.4.0 using the functions detailed under code availability below. Further statistical methodological details are provided in the Supplementary Methods.

### Reporting summary

Further information on research design is available in the Nature Research Reporting Summary linked to this article.

## Supplementary information

Supplementary Information

Reporting Summary

## Data Availability

The authors declare that all data supporting the findings of this study are available within the article and its Supplementary Information files, or are available from the authors upon reasonable request.

## Code availability

Computer codes used to produce results in this article were based on standard R functions. When comparing means across groups we used *t.test* and *oneway.test*. When estimating correlations, we used the *cor* function. Tertiles were estimated through the *quantile* function. Adjustment of *p*-values using Benjamin-Hochberg approach was based on the *p.adjust* function. All the previous functions were in the standard Stat package of R. Survival analysis was based on functions found in the survival package: *survfit*, *survdiff*, *coxph*, and *cox.zph*. Mixed models were fit using function from the Nlme package: the *lme* function was used to fit models and the *intervals* function was used to estimate 95% CI of these models. Finally, hypothesis test in mixed models involved testing hypothesis of specific linear combination of coefficients using the *glht* function from the Multicomp package.
