# Supplementary material for: Concentration and avidity of antibodies to different circumsporozoite epitopes correlate with RTS,S/AS01E malaria vaccine efficacy
Source: Nat Commun. 2019 May 15;10:2174. doi: 10.1038/s41467-019-10195-z (PMC6520358; doi:10.1038/s41467-019-10195-z)
Supplement: Supplementary file 1 — Supplementary Information [file 41467_2019_10195_MOESM1_ESM.pdf]

Concentration and avidity of antibodies to different circumsporozoite epitopes correlate with RTS,S/AS01E malaria vaccine efficacy

Dobaño et al.

Supplementary Information

Supplementary Figure 1

a

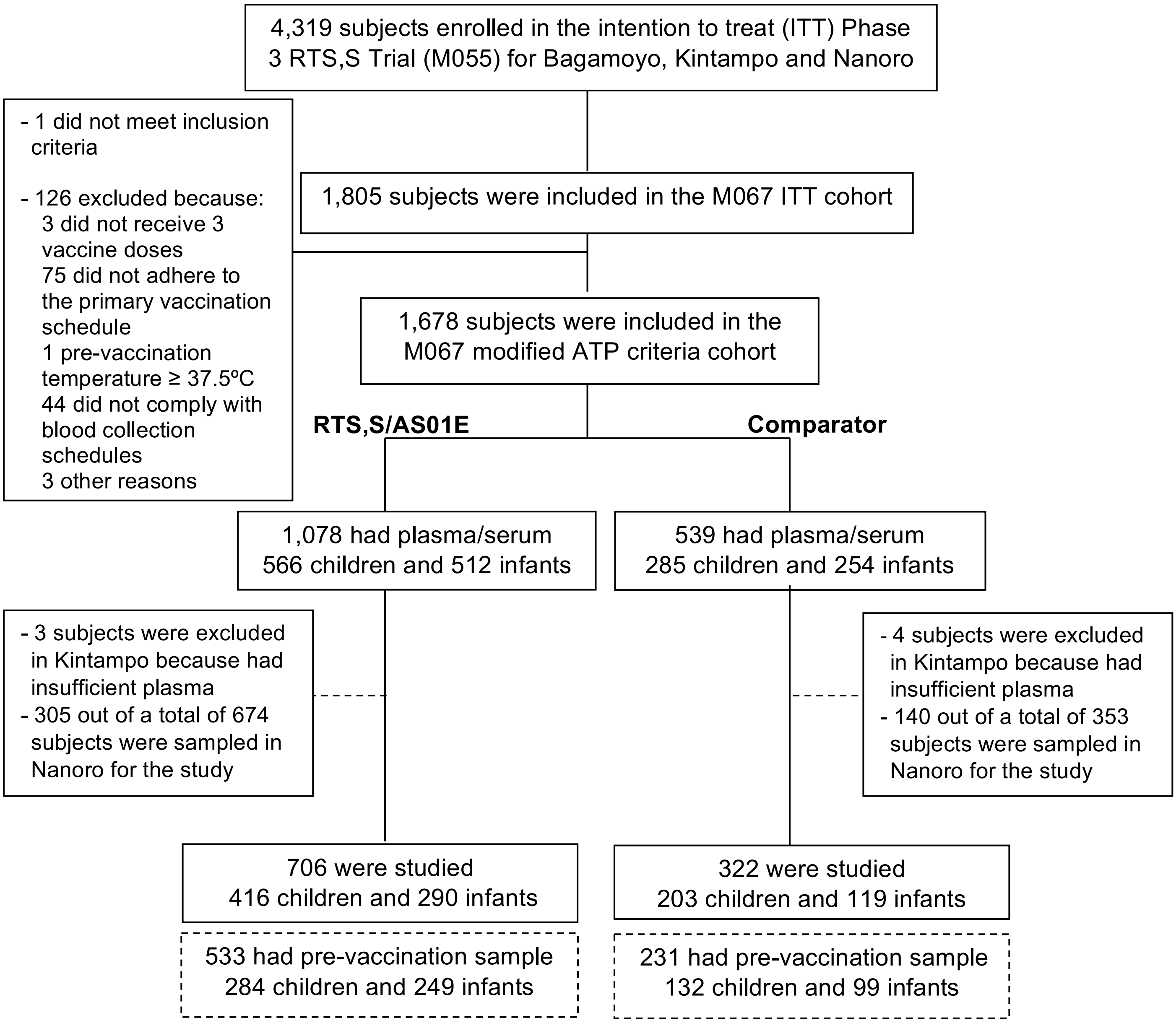

b

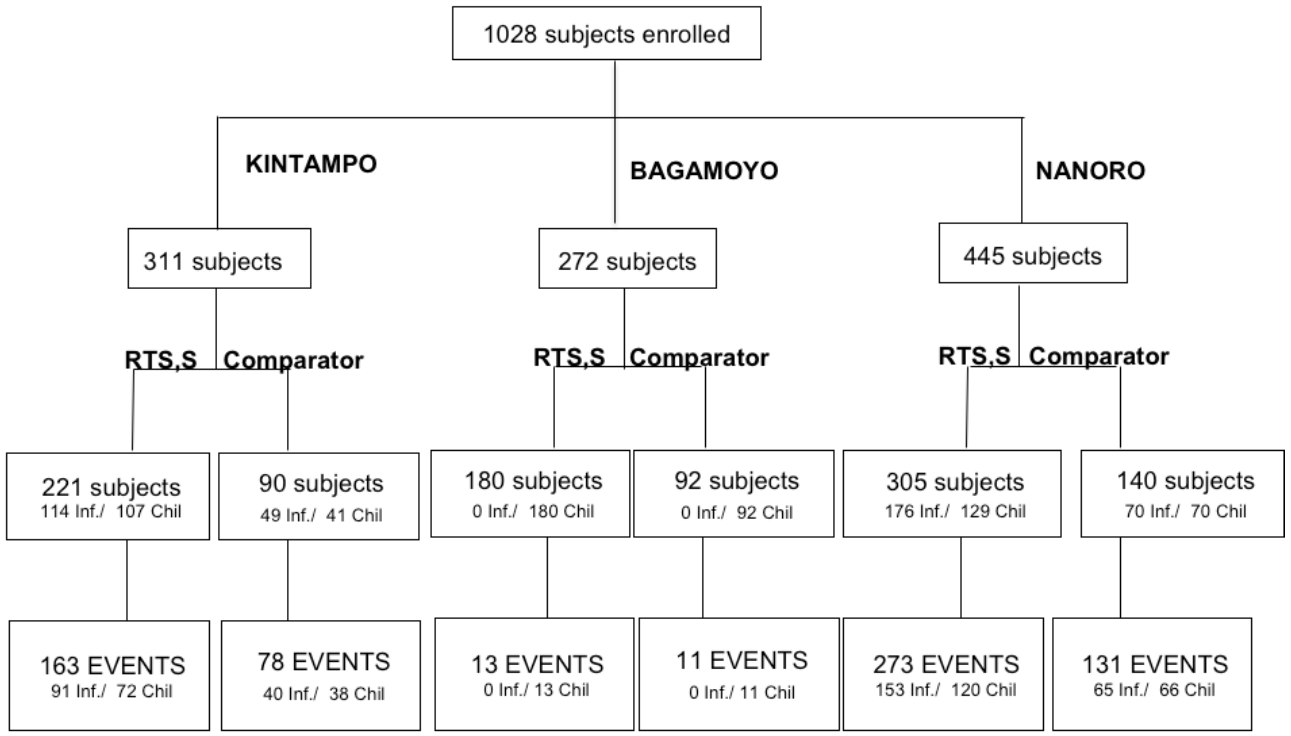

Flowcharts defining eligible, enrolled and the study population after exclusions (a) and distribution of subjects by site indicating number of clinical malaria events (b).

Supplementary Figure 2

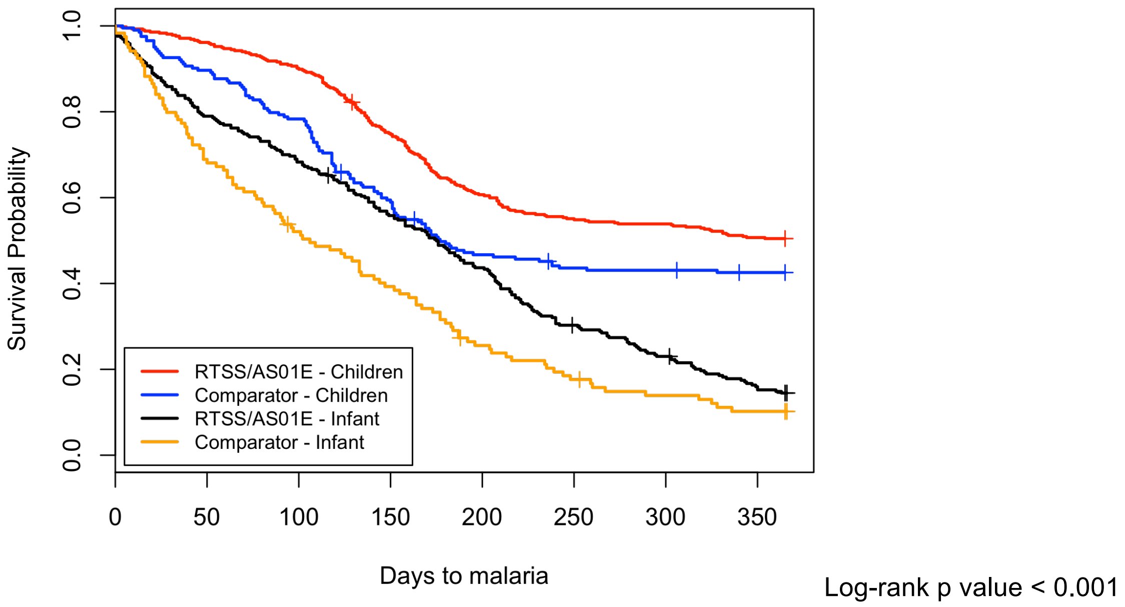

Comparison of Kaplan-Meier survival curves for first clinical malaria events in the study population within the first 12 months of post-vaccination follow-up, stratified by age cohort.

Supplementary Figure 3

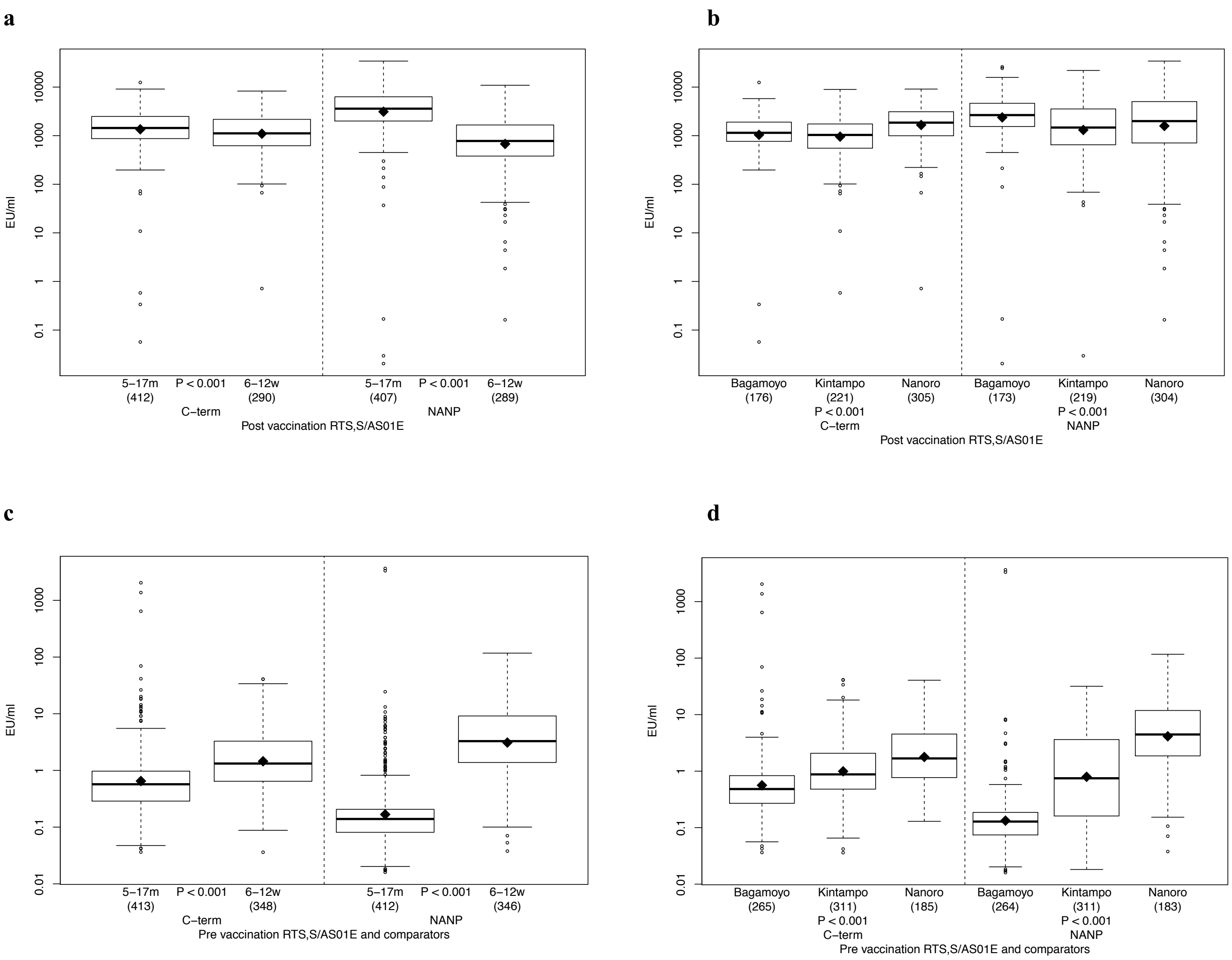

Comparison of CSP NANP and C-terminus IgG concentrations (EU ml^{-1}) between age and sites at post- and pre-vaccination. The solid centre line in the boxplot represents the median and the diamond the geometric mean, the bounds of the box the 25th and 75th interquartile ranges, whiskers display the 1.5 interquartile ranges, and dots the outliers. **a** IgG concentrations after vaccination with RTS,S/AS01E by age cohort. **b** IgG concentrations after vaccination with RTS,S/AS01E by site. **c** IgG concentrations before vaccination by age cohort. **d** IgG concentrations before vaccination by site. Groups were compared by t-tests.

Supplementary Figure 4

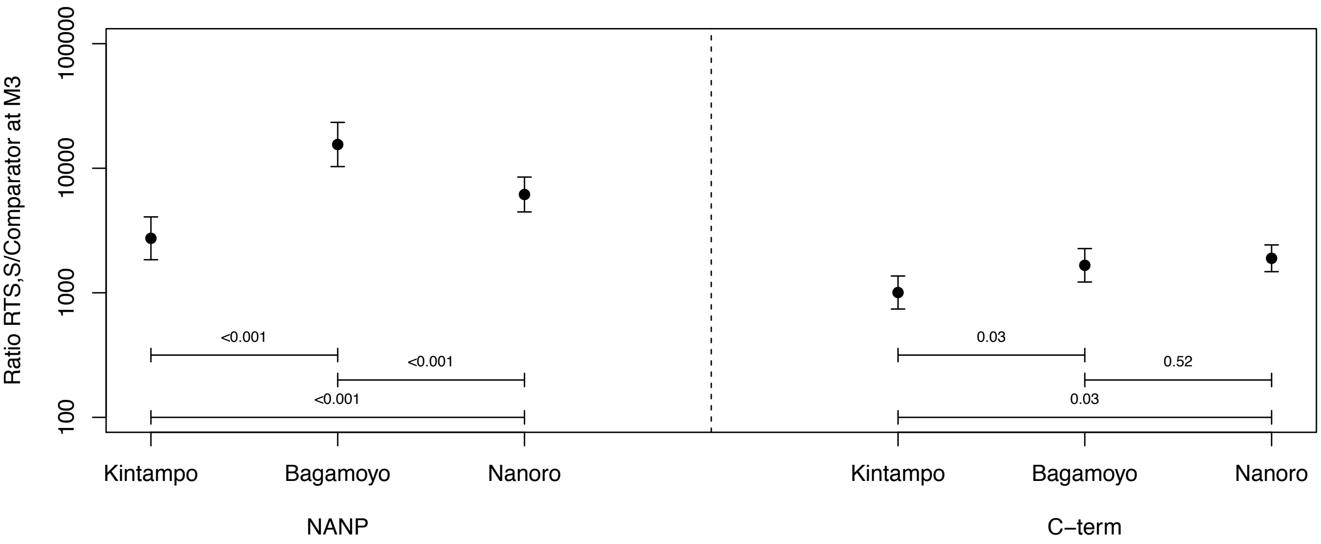

Post-vaccination adjusted increase in NANP and C-terminus CSP IgG concentrations induced by RTS,S/AS01E as compared to comparator vaccinees. The increase of IgG is adjusted by the effect of variables that impacted antibody concentrations (significant in the interaction with vaccination). Adjusted p-values comparing significance of logarithm of RTS,S/Comparator ratios were estimated through mixed models. Error bars represent 95% confidence intervals. M=month.

Supplementary Figure 5

a

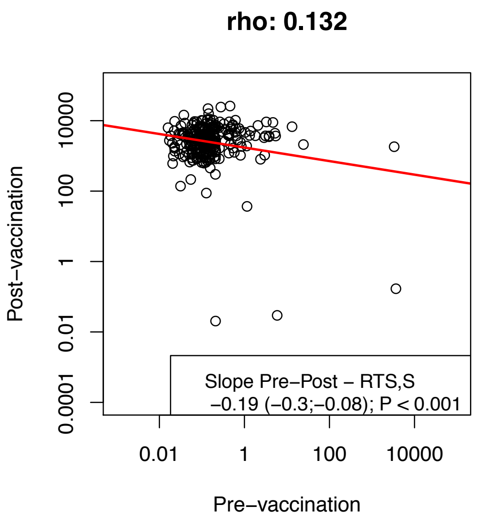

b

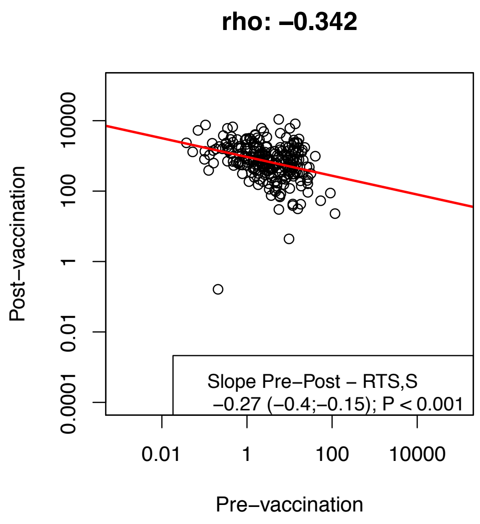

c

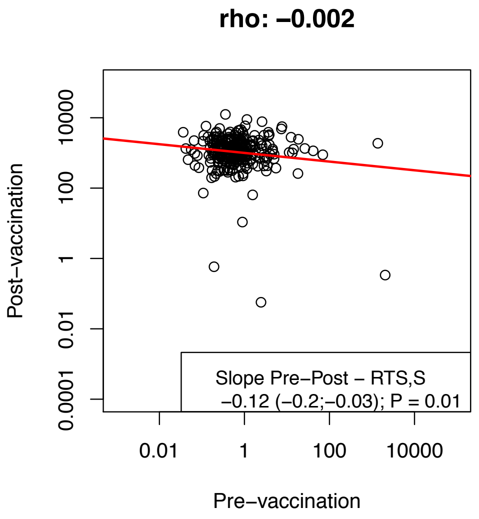

d

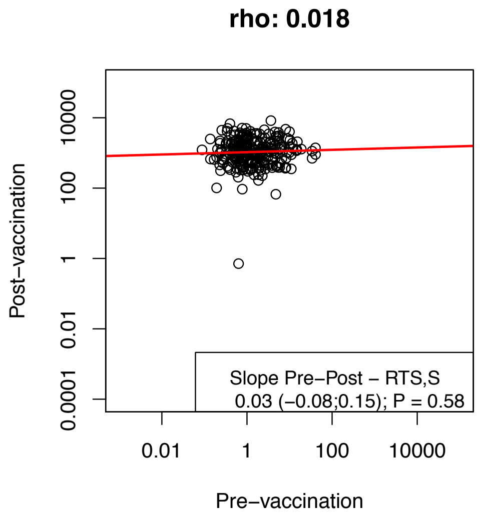

e

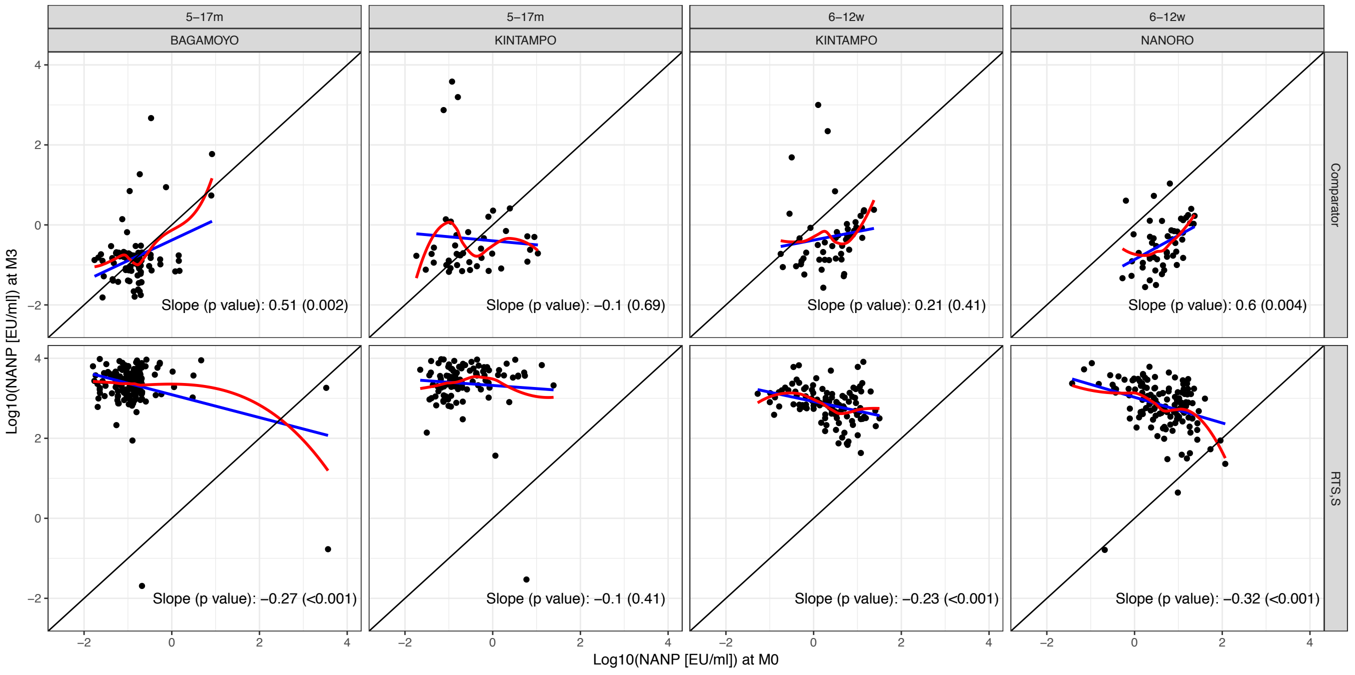

f

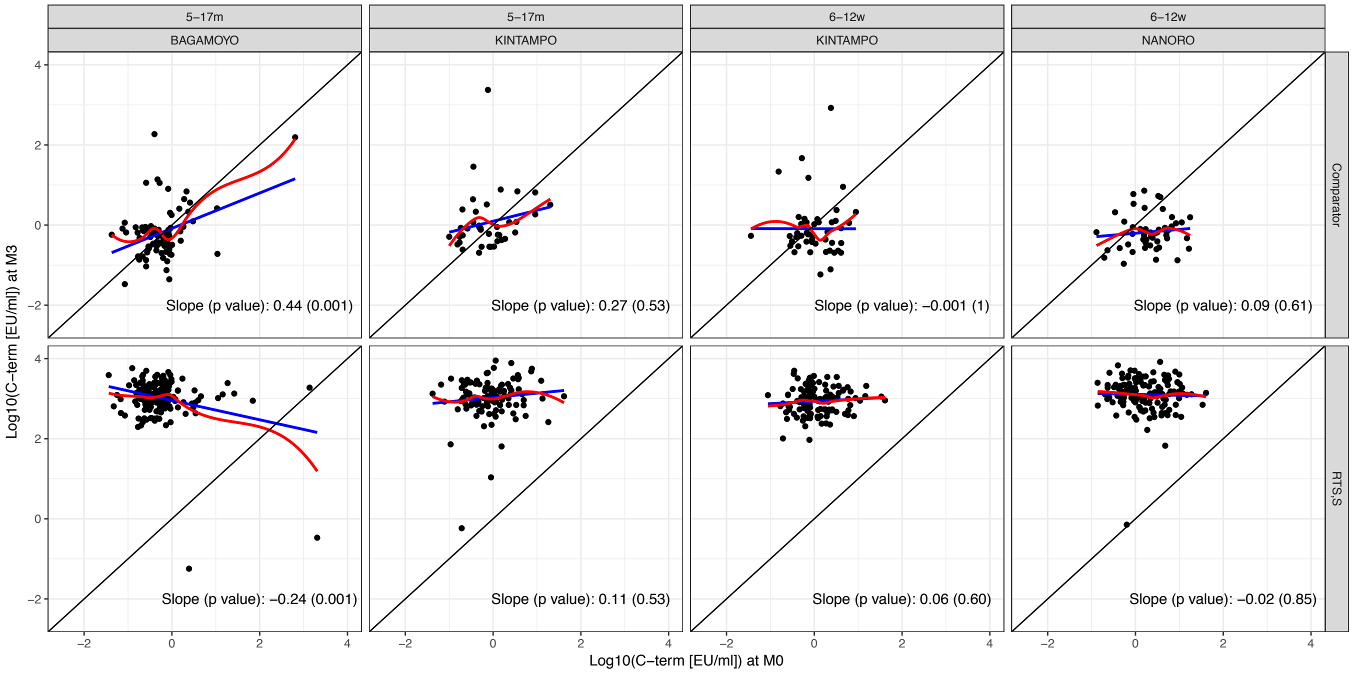

5–17m
BAGAMOYO
5–17m
KINTAMPO
6–12w
KINTAMPO
6–12w
NANORO
Comparator
RTS,S
Slope (p value): 0.44 (0.001)
Slope (p value): 0.27 (0.53)
Slope (p value): −0.001 (1)
Slope (p value): 0.09 (0.61)
Slope (p value): −0.24 (0.001)
Slope (p value): 0.11 (0.53)
Slope (p value): 0.06 (0.60)
Slope (p value): −0.02 (0.85)
Log10(C−term [EU/ml]) at M3
Log10(C−term [EU/ml]) at M0

g

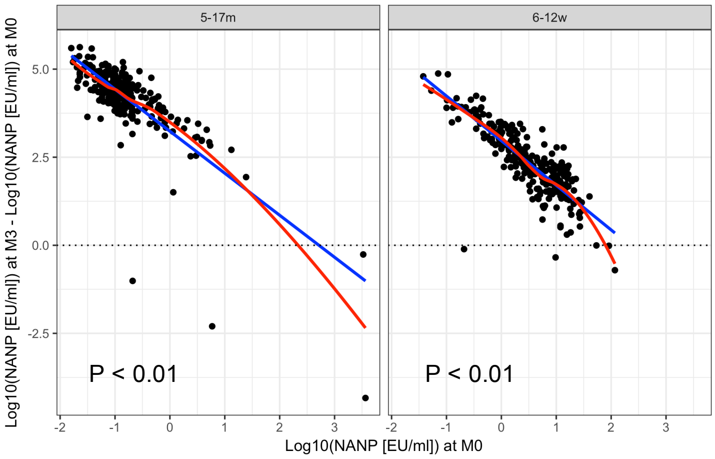

5-17m
6-12w
P < 0.01
P < 0.01
Log10(NANP [EU/ml]) at M3 - Log10(NANP [EU/ml]) at M0
Log10(NANP [EU/ml]) at M0

h

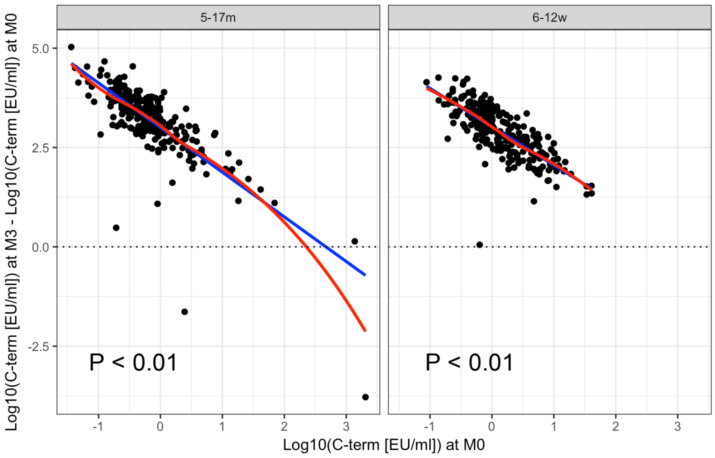

5-17m
6-12w
P < 0.01
P < 0.01
Log10(C-term [EU/ml]) at M3 - Log10(C-term [EU/ml]) at M0
Log10(C-term [EU/ml]) at M0

Correlation analysis between baseline (M0) and post-vaccination (M3) IgG concentrations (log_{10}-transformed EU mL^{-1}) and between M0 and M3-M0 difference in IgG concentrations. In all subjects, M0 vs M3 correlations: **a** CSP NANP, 5-17 months old children (n = 276 matched M0 and M3); **b** CSP NANP, 6-12 weeks old infants (n = 246); **c** CSP C-terminus (C-term), 5-17 months old children (n = 279); **d** CSP C-term, 6-12 weeks old infants (n = 249), with the Spearman correlation coefficients (rho). Stratified by age (children age 5-17 months and infants age 6-12 weeks), site and vaccination status, M0 vs M3 correlations: **e** CSP NANP; **f** CSP C-term, with linear regression (blue line), non-parametric LOESS estimation (red line) and diagonal line (y=x) (black solid line). Stratified by age in RTS,S vaccinees, M0 vs M3-M0 correlations: **g** CSP NANP; **h** CSP C-term, with simple linear regressions with the strength (coefficient for the slope of the line) and significance of associations shown in the figures as well as the correlation coefficients (rho).

The distribution of subjects with both time points was compared with those with only one (M0 or only M3). 272 (26.4%) out of all participants had only one visit sample; 265 (97.4%) had only M3, and 7 (2.57%) only M0. When comparing these subjects with the ones with both time points, no statistically significant differences were found with respect vaccination status (p = 0.408) and concentration values at M0 (p=0.076) overall for both antigens. With regards age cohort (p <0.001), site (p <0.001), and M3 concentration values (p<0.001), statistically differences were found, being those with only one visit older, mainly from Bagamoyo, and with higher M3 concentration.

Supplementary Figure 6

a CSP NANP IgG

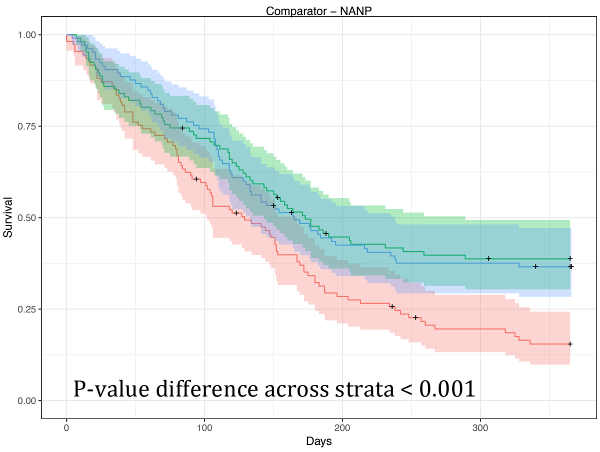

Median survival time (days):
High: 129 (102, 167)
Medium: 165 (133, 238)
Low: 173 (143, 289)

b CSP C-terminus IgG

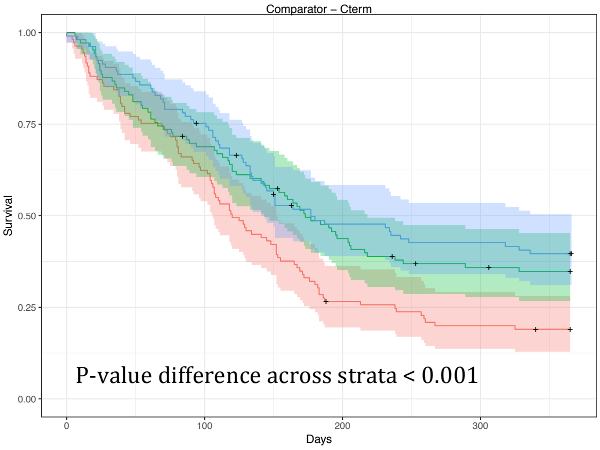

Median survival time (days):
High: 120 (106, 152)
Medium: 289 (141, ∝)
Low: 173 (143, 218)

c HBsAg IgG

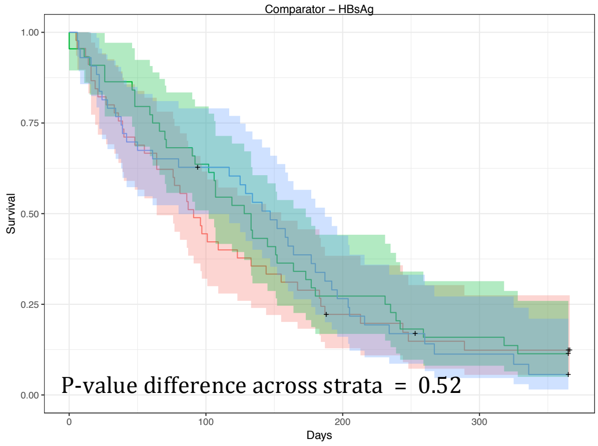

Median survival time (days):
High: 91 (64, 155)
Medium: 147 (80, 187)
Low: 130 (102, 173)

Kaplan-Meier estimates of time to the first clinical malaria episode during the 12-month follow-up period after the third vaccine dose by the three tertiles (high in red; medium in blue; low in green) of IgG concentrations (EU mL^{-1}) to NANP (a), to C-term (b) and to HBsAg (c) at month 3 in comparator vaccinees. The highest tertile included the positivity threshold. The plot legends show the median survival time (days), i.e., time at which the survivorship function equals 0.5, for each antibody tertile with the corresponding 95% confidence interval and the p-values assessing differences in the distribution of survival time across the three strata in each of the four subgroups (estimated through the Log-rank test).

Supplementary Figure 7

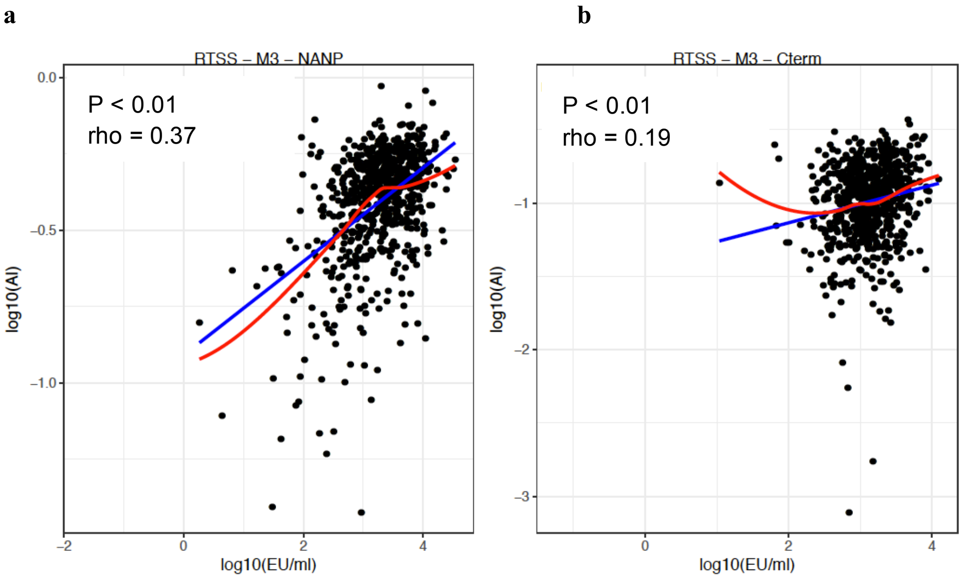

Correlation between concentration (log_{10}EU mL^{-1}) and avidity (log_{10}AI) for NANP (a) and C-terminus (C-term) (b) CSP antigens at month 3 (M3) post-vaccination. Strength of association, p-values and Spearman correlation coefficients are shown.

Supplementary Figure 8

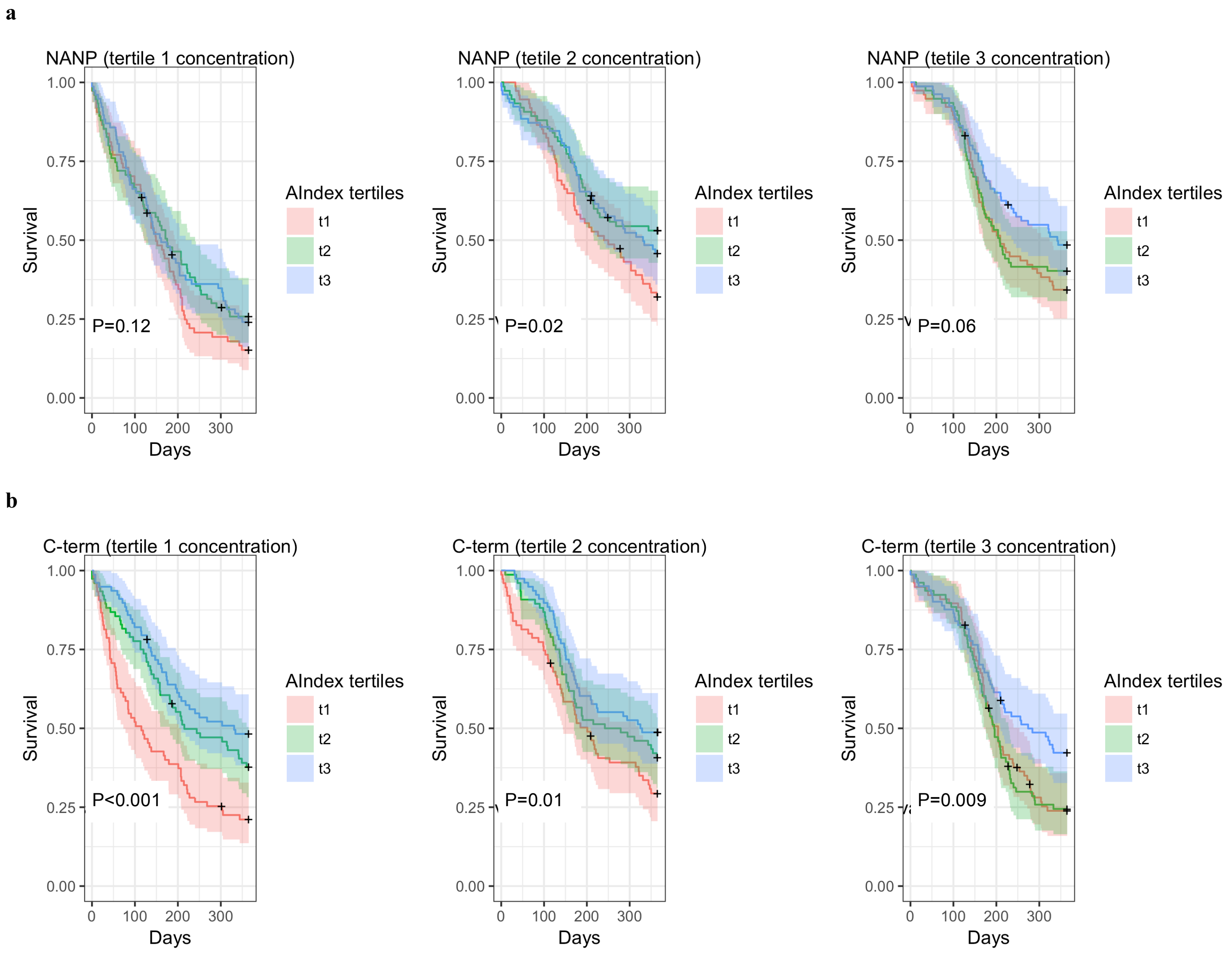

Correlation with protection from first clinical malaria episode of Avidity Index (AI) given IgG concentration of CSP NANP (a) and C-term (b). Kaplan-Meier curves with IgG (log_{10}EU mL^{-1}) and AI (log_{10}AI) stratified in tertiles (t3 = highest, t2 = moderate, t3 = lowest). P-values were obtained through log-rank tests.

Supplementary Table 1.

	NANP	C-term
**Age Group, *n (%)***		
5-17 months	404 (98%)	409 (99%)
6-12 weeks	289 (99.7%)	289 (99.7%)
P-value	0.06	0.42
**Study site, *n (%)***		
Bagamoyo	171 (96%)	174 (98%)
Kintampo	219 (99.1%)	220 (99.5%)
Nanoro	303 (99.3%)	304 (99.7%)
P-value	0.02	0.08

Seropositivity stratified by age and site in RTS,S/AS01E vaccinees (n=704). Number and proportion of samples whose CSP IgG concentrations (EU mL^{-1}) were above the assay positivity thresholds provided by IAVI-HIL (NANP > 1.78 EU mL^{-1} and C-term > 2.95 EU mL^{-1}). Only post-vaccination (M3) data are included. P-values were estimated through Pearson Chi-square tests.

Supplementary Table 2

a Univariable models

	RTS,S/AS01E vaccinees at month 3				Change from month 0 to 3 (M3/M0 ratio in log_{10} scale) in RTS,S/AS01E vaccinees	
	Concentration		Avidity Index		Concentration	
	NANP	C-term	NANP	C-term	NANP	C-term
**Age cohort**						
5-17 months	Ref.	Ref.	Ref.	Ref.	Ref.	Ref.
6-12 weeks	**0.22 (0.17;0.28)**	0.83 (0.68;1.00)	**0.74 (0.68;0.79)**	**0.55 (0.50;0.62)**	**0.01 (0.01;0.02)**	**0.37 (0.27;0.51)**
**Site**						
Bagamoyo	Ref.	Ref.	Ref.	Ref.	Ref.	Ref.
Kintampo	**0.66 (0.47;0.93)**	0.87 (0.69;1.12)	**0.87 (0.79;0.95)**	0.87 (0.74;1.00)	**0.15 (0.08;0.25)**	**0.49 (0.33;0.71)**
Nanoro	**0.68 (0.49;0.91)**	1.55 (1.26;1.96)	**0.83 (0.76;0.91)**	**0.79 (0.69;0.89)**	**0.03 (0.02;0.05)**	**0.57 (0.39;0.84)**
**Sex**						
Female	Ref.	Ref.	Ref.	Ref.	Ref.	Ref.
Male	**0.74 (0.58;0.98)**	0.87 (0.72;1.05)	1.02 (0.93;1.10)	1.1 (0.98;1.23)	0.65 (0.38;1.12)	0.78 (0.56;1.07)
**Previous episodes**	1.02 (0.76;1.38)	**1.41 (1.12;1.74)**	0.93 (0.85;1.02)	0.89 (0.78;1.00)	**0.29 (0.15;0.55)**	**0.81 (0.54;1.21)**
**Season malaria transmission**						
High	Ref.	Ref.	Ref.	Ref.	Ref.	Ref.
No-Low	**1.55 (1.20;2.04)**	**1.45 (1.20;1.74)**	1.07 (1.00;1.15)	1.1 (0.98;1.23)	**2.18 (1.25;3.79)**	**1.59 (1.14;2.22)**
**WAZ**	**0.76 (0.68;0.85)**	**0.83 (0.78;0.91)**	0.98 (0.95;1.02)	0.95 (0.91;1.00)	**0.72 (0.57;0.91)**	**0.76 (0.66;0.87)**
**HAZ**	1.0 (0.89;1.1)	**0.91 (0.85;0.98)**	1.02 (1.0;1.05)	1.02 (0.98;1.07)	**1.40 (1.16;1.70)**	0.94 (0.84;1.06)
**Haemoglobin**	**0.78 (0.72;0.83)**	0.95 (0.91;1.02)	**0.95 (0.93;0.98)**	**0.91 (0.89;0.95)**	**0.50 (0.43;0.59)**	**0.85 (0.77;0.95)**
**M0 IgG levels**	**0.48 (0.41;0.56)**	0.87 (0.72;1.05)	1.23 (1.10;1.35)	1.15 (0.98;1.35)	--	--

b Multivariable models

	RTS,S/AS01E vaccinees at month 3				Change from month 0 to 3 (M3/M0 ratio in log_{10} scale) in RTS,S/AS01E vaccinees	
	Concentration		Avidity Index		Concentration	
	NANP	C-term	NANP	C-term	NANP	C-term
**Main Model**						
**Age** (6-12 weeks)	**0.17 (0.14;0.22)**	**0.65 (0.54;0.76)**	**0.74 (0.69;0.79)**	**0.51 (0.47;0.56)**	**0.02 (0.01;0.03)**	**0.46 (0.32;0.67)**
**Study Site**						
Bagamoyo	Ref.	Ref.	Ref.	Ref.	Ref.	Ref.
Kintampo	**1.35 (1.02;1.82)**	1.15 (0.93;1.45)	0.98 (0.91;1.07)	**1.15 (1.02;1.29)**	0.79 (0.49;1.29)	0.75 (0.51;1.10)
Nanoro	**1.82 (1.38;2.40)**	**2.04 (1.66;2.57)**	1.00 (0.91;1.01)	**1.17 (1.05;1.32)**	0.62 (0.34;1.10)	0.99 (0.61;1.61)
**Adjusted Model**						
**M0 IgG levels**	**0.58 (0.47;0.72)**	--	--	--	--	--
**Age** (6-12 weeks)	**0.56 (0.34;0.93)**	0.87 (0.58;1.35)	**0.65 (0.55;0.78)**	**0.41 (0.32;0.52)**	**0.02 (0.01;0.05)**	**0.50 (0.31;0.80)**
**Study Site**						
Bagamoyo	Ref.	Ref.	Ref.	Ref.	Ref.	Ref.
Kintampo	1.10 (0.72;1.66)	0.72 (0.46;1.12)	1.05 (0.87;1.23)	1.29 (0.98;1.66)	**0.47 (0.26;0.86)**	**0.59 (0.39;0.90)**
Nanoro	1.10 (0.62;2.00)	1.29 (0.76;2.14)	1.12 (0.91;1.35)	1.32 (1.00;1.78)	**0.42 (0.21;0.86)**	0.86 (0.48;1.54)
**Sex**						
Female	Ref.	Ref.	Ref.	Ref.	Ref.	Ref.
Male	0.78 (0.59;1.00)	0.85 (0.68;1.07)	--	**1.23 (1.07;1.38)**	0.71 (0.48;1.06)	0.78 (0.58;1.06)
**Season malaria transmission**	--	--	--	--	--	
High	Ref.	Ref.	Ref.	Ref.	Ref.	Ref.
Low or no	--	--	--	--	0.65 (0.39;1.06)	--
**WAZ**	**0.81 (0.72;0.91)**	--	--	--	**0.83 (0.70;0.98)**	**0.80 (0.70;0.92)**
**Haemoglobin**	--	--	--	1.05 (1.00;1.10)	0.90 (0.79;1.04)	--

Factors affecting RTS,S/AS01E immunogenicity. Relevant demographic, clinical and epidemiological factors associated with vaccine-induced IgG concentration (EU mL^{-1}) measured in log_{10} scale. Coefficients and 95% confidence intervals (CI). In bold font values that are statistical significance based on 95% CI not including zero. Previous episodes refer to having had clinical malaria episodes during the vaccination period (between month 0 [M0] and M3). Season of malaria transmission refers to the period when the post-vaccination sample was collected. WAZ: weight-for-age Z-score; HAZ: height-for-age Z-score.

Supplementary Table 3

	First clinical malaria episode		Recurrent clinical malaria episodes	
	**CSP NANP**	**CSP C-term**	**CSP NANP**	**CSP C-term**
**$Log_{10}(EU\ mL^{-1})$**	1.00 (0.84;1.20)	0.98 (0.79;1.23)	1.05 (0.96;1.16)	1.08 (0.94;1.24)
**Site**				
Bagamoyo	Ref.	Ref.	Ref.	Ref.
Kintampo	16.4 (8.13;33.3)	16.7 (8.27;33.9)	12.6 (6.23;25.4)	13.0 (6.42;26.5)
Nanoro	--	--	15.1 (7.56;30.3)	15.4 (7.67;31.0)
<180 days	5.52 (2.24;13.6) * $[1.01\ (1.01;1.02)]^{Days}$¶	5.69 (2.30;14.1) * $[1.01\ (1.01;1.02)]^{Days}$	--	--
≥180 days	2,202 (21.5;225729) * $[0.98\ (0.96;1.00)]^{Days}$	2,228 (21.8;228144) * $[0.98\ (0.96;1.01)]^{Days}$	--	--
**Age cohort**				
5-17 months	Ref.	Ref.	Ref.	Ref.
6-12 weeks	--	--	0.93 (0.82;1.07)	0.95 (0.83;1.08)
<180 days	2.16 (1.20;3.86) * $[0.99\ (0.98;0.99)]^{Days}$	2.12 (1.18;3.80) * $[0.99\ (0.98;0.99)^{Days}$	--	--
≥180 days	0.05 (0.001;2.05) * $[1.01\ (1.00;1.03)]^{Days}$	0.04 (0.001;2.03) * $[1.01\ (1.00;1.03)]^{Days}$	--	--

Hazard ratios (HR) and 95% confidence intervals (CI) for clinical malaria during the 12-month follow-up period after the third RTS,S/AS01E dose for the anti-CSP NANP and C-terminus (C-term) IgG models in comparator vaccinees at month 3, including: IgG concentration (log_{10} EU mL^{-1}), site and age. Proportionality of hazards was assessed by the scaled Schoenfeld residuals and time-varying coefficients were included if necessary to hold the proportionality of hazards assumption.

Ref. means the reference variable with which age cohort and site are compared.

¶This equation shows the relation of HR as a function of time. Within parentheses the 95% CI. The equation without CI would be: 5.52 * 1.01^days. This means the risk in Nanoro over Bagamoyo is 5x higher at day1 but this risks increases with time up to 180 days. Below, at 180 days Nanoro has more risk than Bagamoyo, but the magnitude decreases because the risk at 180 days (2,202) decreases having days into account at a rate of 0.98. I.e. at 180 days the HR is 2,202 * 0.98^180.

Supplementary Table 4

a.1) NANP CSP – M0

M0		RTS,S (n=533)			Comparator (n = 231)	
	Bagamoyo (n=177)	Kintampo (n=221)	Nanoro (n=135)	Bagamoyo (n=91)	Kintampo (n=90)	Nanoro (n=135)
5-17m	170	95	--	84	33	--
6-12w	--	42	27	--	15	6

a.2) NANP CSP – M3

M3		RTS,S (n=704)			Comparator (n = 322)	
	Bagamoyo (n=178)	Kintampo (n=221)	Nanoro (n=305)	Bagamoyo (n=92)	Kintampo (n=90)	Nanoro (n=140)
5-17m	2	1	2	84	35	63
6-12w	--	1	1	--	40	60

b.1) C-term CSP – M0

M0		RTS,S (n=533)			Comparator (n = 231)	
	Bagamoyo (n=177)	Kintampo (n=221)	Nanoro (n=135)	Bagamoyo (n=91)	Kintampo (n=90)	Nanoro (n=135)
5-17m	160	89	--	87	35	--
6-12w	--	92	90	--	39	26

b.2) C-term CSP – M3

M3		RTS,S (n=704)			Comparator (n = 322)	
	Bagamoyo (n=178)	Kintampo (n=221)	Nanoro (n=305)	Bagamoyo (n=92)	Kintampo (n=90)	Nanoro (n=140)
5-17m	2	1	0	83	32	60
6-12w	--	0	1	--	44	61

Distribution of non-quantitated samples across comparison groups of interest.
Proportion of month 3 (M3) non-quantitated samples by vaccination status, age, site. Same for M0.

Supplementary Table 5

a NANP CSP

		ELISA samples		
		**Quantitated**	**Non-Quantitated**	**Overall**
**ELISA chaotropic agent samples**	**Quantitated**	--	--	(0.70/0.67)
	**Non-Quantitated**	0.61/0.60	0.68/0.50	

b C-terminal CSP

		ELISA samples		
		**Quantitated**	**Non-Quantitated**	**Overall**
**ELISA chaotropic agent samples**	**Quantitated**	--	--	(0.90/0.91)
	**Non-Quantitated**	0.49/0.44	0.55/0.49	

Evaluating the adherence of imputation to the original distribution of data.
Spearman correlation comparison between optical densities before and after imputation (Before/After) of the non-quantitated samples by antigen. Correlations were calculated according to the characteristics of the imputation procedure, i.e., depending on the combination of the ELISA sample with its correspondent with chaotropic agent.

Supplementary Methods

Subject and sample selection. Individuals considered eligible for the study were those from the according to protocol (ATP) MAL067 cohort who had 75 µl of M3 plasma/serum sample stored. The ATP criteria included receiving 3 doses of vaccine, and having a blood sample collected approximately 30 days after vaccination (M3). In Kintampo (n= 311, 148 children and 163 infants) and Bagamoyo (n=272 children) all eligible children were selected, and in Nanoro, 445 participants (199 children and 246 infants) were randomly selected (Table 1 and Supplementary Figure 1). Nearly all subjects in Kintampo (100%) and Bagamoyo (99%) had pre-vaccination (M0) samples available, while in Nanoro, only 41% subjects, all infants, had M0 samples.

Subject follow up. Subjects were followed up by passive case detection (PCD) starting 14 days from the date of sample collection to ascertain that antibody responses measured would be predictive of a future malaria episode and not markers of past malaria infection. The follow-up for clinical malaria events in this study was defined for the subsequent 12 months, during which subjects were visited monthly to also assess whether they were still living in the study area and participating in the study. After the 12 months of follow up, subjects were censored. Reasons for early termination were recorded.

ELISA data pre-processing. ELISA data were captured using Magellan software (TECAN, Switzerland). The standard curves were estimated using a 4-parameter logistic (4PL) regression and were fitted after averaging the optical density (OD) of the standard points replicates. To provide EU mL^{-1} data in the absence of an international standard, the curves were pre-calibrated at IAVI-HIL by determining an average reciprocal of dilution for each monoclonal antibody that provided an OD=1 (12,114 EU mL^{-1} and 6,966 EU mL^{-1} for CSP C-term and NANP, respectively). Sample IgG concentrations (EU mL^{-1}) were interpolated from the 4PL standard curve using the OD of the lowest dilution in the linear section of the curve (under the upper limit of detection). The replicates of the test samples were averaged and used as the test sample measurement.

Quality control for each plate was based on positive, negative, and blank controls. A plate was considered to fail based on: i) thresholds for blank and negative controls and Westgard rules for positive controls; ii) expected standard curve characteristics. A sample was considered to fail based on: i) replicates variability; ii) high IgG titre requiring additional dilution to fall on the linear section of the 4PL curve (above 1:7,200).

Using a uniform distribution, the non-quantitated ELISA concentration data were imputed using values between limit of detection (LOD) and limit of quantification (LOQ), provided by IAVI-HIL. To account for the correlation between avidity concentration and ELISA concentration data, the imputed values of the avidity samples were generated given the ELISA values, imputed or observed (whether quantitated or not) by fitting a model with the OD of the non-quantitated samples. This model was fitted using generalized least squares models (GLS). Using the original ODs we maintained the correlation structure between ELISA and avidity data, and by fitting a GLS model we accounted for the heteroscedasticity of the data. The avidity ODs were fitted using as a predictor the OD of the imputed (or observed) ELISA after converting the imputed (or observed) concentration in OD (using a randomly selected standard curve from all assayed plates). To convert the imputed avidity OD into concentration we used the same standard curve used to convert imputed ELISA concentrations to ODs. The GLS models were antigen-specific, type of non-quantitated data-specific (i.e., whether it was avidity or not) and were validated by inspecting the residuals distribution and comparing the correlation structure between the original OD of the data and the imputed one (Supplementary Tables 4 and 5). Tables contain information on how many values were imputed and how many measured.

Immunogenicity data analysis. Mixed linear models were fit including as outcomes M3 and M0 anti-NANP and M3 and M0 anti-C-term CSP IgG concentrations, as well as vaccination, time point, and the appropriate interaction. Necessity of including a random slope for changes over time was assessed during model building through exploratory analysis (trajectory plots) and statistical tests, i.e., ANOVA test comparing two models: i) those with random intercept and random slope, and ii) those with only random intercept. The main mixed model equation, without any other adjusted covariate, used in the analysis was expressed as follows:

$$Concentration \sim \beta_0 + \beta_1 * M3 + \beta_2 * RTS,S + \beta_3 * M3\ x\ RTS,S + RE(Visit/Subject)$$

Where $RE(Visit/Subject)$ defines the random intercepts per subject and random slope M3-M0. Using this notation we were able to estimate the concentration, for example, for the M3-RTS,S cohort as ($\beta_0 + \beta_1 + \beta_2 + \beta_3$) and for the M0-RTS,S cohort as ($\beta_0 + \beta_2 + \beta_3$), being the concentration difference between both timepoints M3-M0 for RTS,S cohort equal to β_1.

After linearity of associations with continuous covariates was evaluated, parametric generalized linear models (GLM) were fit and non-linear terms were included as b-splines with degrees of freedom estimated for the spline term in the generalized additive models (GAM) model[1]. In models including relevant covariates, an adjusted effect of vaccination was evaluated and the impact of these covariates on immunogenicity was assessed through interaction terms. Covariates were retained in models based on statistical significance association with outcomes, and on their impact on the correlate coefficients.

Correlates of protection data analysis. The number of events between M0 and M3 and after M3 was estimated for the two vaccination and age groups of each study site and compared across vaccination groups in crude and stratified analyses through Poisson regression. Kaplan-Meier curves comparing time to first malaria event through log-rank tests were also estimated within each vaccination group (RTS,S and comparators) for CSP IgG positive vs negative subjects.

Supplementary References

1. Hastie T, Tibshirani R. Generalized additive models for medical research. *Stat Methods Med Res* **4**, 187-196 (1995).
