# Supplementary material for: Concentration and avidity of antibodies to different circumsporozoite epitopes correlate with RTS,S/AS01E malaria vaccine efficacy
Source: Nat Commun. 2019 May 15;10:2174. doi: 10.1038/s41467-019-10195-z (PMC6520358; doi:10.1038/s41467-019-10195-z)
Supplement: Supplementary file 2 — Reporting Summary [file 41467_2019_10195_MOESM2_ESM.pdf]

nature research

Corresponding author(s): Carlota Dobaño NCOMMS-18-15674C

Last updated by author(s): Apr 22, 2019

Reporting Summary

Nature Research wishes to improve the reproducibility of the work that we publish. This form provides structure for consistency and transparency in reporting. For further information on Nature Research policies, see Authors & Referees and the Editorial Policy Checklist .

Statistics

For all statistical analyses, confirm that the following items are present in the figure legend, table legend, main text, or Methods section.

n/a	Confirmed	
☐	☒	The exact sample size (n) for each experimental group/condition, given as a discrete number and unit of measurement
☐	☒	A statement on whether measurements were taken from distinct samples or whether the same sample was measured repeatedly
☐	☒	The statistical test(s) used AND whether they are one- or two-sided<br>*Only common tests should be described solely by name; describe more complex techniques in the Methods section.*
☐	☒	A description of all covariates tested
☐	☒	A description of any assumptions or corrections, such as tests of normality and adjustment for multiple comparisons
☐	☒	A full description of the statistical parameters including central tendency (e.g. means) or other basic estimates (e.g. regression coefficient) AND variation (e.g. standard deviation) or associated estimates of uncertainty (e.g. confidence intervals)
☐	☒	For null hypothesis testing, the test statistic (e.g. F, t, r) with confidence intervals, effect sizes, degrees of freedom and P value noted<br>*Give P values as exact values whenever suitable.*
☒	☐	For Bayesian analysis, information on the choice of priors and Markov chain Monte Carlo settings
☒	☐	For hierarchical and complex designs, identification of the appropriate level for tests and full reporting of outcomes
☐	☒	Estimates of effect sizes (e.g. Cohen's d, Pearson's r), indicating how they were calculated

Our web collection on statistics for biologists contains articles on many of the points above.

Software and code

Policy information about availability of computer code

Data collection

GSK transfered to ISGlobal the clinical data from the parental trial MAL055, collected as published by the vaccine trial sponsor. ELISA antibody data was generated following IAVI-HIL reference laboratory Standard Operating Procedures and transfered to ISGlobal by RedCap.

Data analysis

Computer codes used to produce results in this article were based on standard R functions. When comparing means across groups we used t.test and oneway.test. When estimating correlations, we used the cor function. Tertiles were estimated through the quantile function. Adjustment of p-values using Benjamin-Hochberg approach was based on the p.adjust function. All the previous functions were in the standard Stat package of R. Survival analysis was based on functions found in the survival package: survfit, survdiff, coxph, and cox.zph. Mixed models were fit using function from the Nlme package: the lme function was used to fit models and the intervals function was used to estimate 95% CI of these models. Finally, hypothesis test in mixed models involved testing hypothesis of specific linear combination of coefficients using the glht function from the Multicomp package.

For manuscripts utilizing custom algorithms or software that are central to the research but not yet described in published literature, software must be made available to editors/reviewers. We strongly encourage code deposition in a community repository (e.g. GitHub). See the Nature Research guidelines for submitting code & software for further information.

Data

Policy information about availability of data

All manuscripts must include a data availability statement. This statement should provide the following information, where applicable:

- Accession codes, unique identifiers, or web links for publicly available datasets
- A list of figures that have associated raw data
- A description of any restrictions on data availability

The authors declare that all data supporting the findings of this study are available within the article and its Supplementary Information files, or are available from the authors upon reasonable request

Field-specific reporting

Please select the one below that is the best fit for your research. If you are not sure, read the appropriate sections before making your selection.

- [x] Life sciences
- [ ] Behavioural & social sciences
- [ ] Ecological, evolutionary & environmental sciences

For a reference copy of the document with all sections, see nature.com/documents/nr-reporting-summary-flat.pdf

Life sciences study design

All studies must disclose on these points even when the disclosure is negative.

Sample size	The sample size (n=1028 subjects) calculation rationale description is included in the manuscript (Methods)
Data exclusions	No data was excluded; the testing of the ELISA datapoints not passing the QA/QC was repeated
Replication	ELISA experiments included the standard replicates as per Standard Operating Procedures of the IAVI-HIL Reference Lab (UK)
Randomization	The randomization was part of the parental clinical trial, which was a randomized controlled trial (RCT). In addition, serum/plasma samples were randomly distributed accross plates prior to testing to avoid batch effects due to day to day or operator variations
Blinding	The blinding was part of the parental clinical trial which was a double blind RCT. In addition, ELISA operators were blinded as to the study samples.

Reporting for specific materials, systems and methods

We require information from authors about some types of materials, experimental systems and methods used in many studies. Here, indicate whether each material, system or method listed is relevant to your study. If you are not sure if a list item applies to your research, read the appropriate section before selecting a response.

Materials & experimental systems

n/a	Involved in the study	
[ ]	[x]	Antibodies
[x]	[ ]	Eukaryotic cell lines
[x]	[ ]	Palaeontology
[x]	[ ]	Animals and other organisms
[ ]	[x]	Human research participants
[ ]	[x]	Clinical data

Methods

n/a	Involved in the study	
[x]	[ ]	ChIP-seq
[x]	[ ]	Flow cytometry
[x]	[ ]	MRI-based neuroimaging

Antibodies

Antibodies used	ELISA secondary antibody reagents included are described in the IAVI-HIL documentation: "ELISA and avidity assays for assessing anti-CSP IgG antibodies (SLM-0034)" and "ELISA and avidity assays for assessing anti-CSP IgG antibodies (SLM-0034)". Catalog numbers and dilutions of the ELISA assay antibodies are included in the manuscript.
Validation	ELISA assays at the IAVI-HIL reference lab are standardized and all reagents validated

Human research participants

Policy information about studies involving human research participants

Population characteristics	Age and site (location) were the main variables controlled for in the analysis. In addition we took into account other baseline variables: sex, malaria seasonality, nutritional scores, hemoglobin concentration and prior malaria exposure, in the analysis.
Recruitment	Our study did not recruit the volunteers as we did not perform the clinical trial. We analyzed the samples already taken and stored as part of the clinical trial, within a nested substudy (ancillary immunology study to the parental phase 3 RCT) . Recruitment has been explained in the publications of the parental Mal055 phase 3 trial.
Ethics oversight	The study protocol was approved by the following relevant institutional or national Ethics Committees: National Institute for Medical Research, Ifakara Health Institute (IHI IRB), Tanzania; Ethics Committee of the University and State of Basel (EKBB), Switzerland; Kintampo Health Research Centre (KHRC) Institutional Ethics Committee (IEC) and Noguchi Memorial Institute for Medical Research IRB, Ghana; Institutional EC IRSS and Comite d'Ethique pour la Recherche en Sante, Burkina Faso; Comitè Ètic d'Investigació Clínica (CEIC, Hospital Clínic, UB), Barcelona, Spain; Research Ethics Committee (REC), PATH, USA.

Note that full information on the approval of the study protocol must also be provided in the manuscript.

Clinical data

Policy information about clinical studies

All manuscripts should comply with the ICMJEguidelines for publication of clinical research and a completedCONSORT checklist must be included with all submissions.

Clinical trial registration	The parental trial registration in ClinicalTrials.gov is NCT00866619
Study protocol	Supplementary materials from the parental trial (MAL055) publications
Data collection	GSK (sponsor of the MAL055 trial) collected the data in the trial sites involved (Bagamoyo, Nanoro, Kintampo) in 2009-2010 with the procedures as published in the parental trial publications
Outcomes	The primary and secondary outcome measures pre-defined in the parental clinical trial are described in the MAL055 publications. For the immunology ancillary study here, the primary outcome measure for the analysis of association between antibody concentrations or avidity and vaccine-induced protection was clinical malaria without any parasite density threshold (fever and any parasitemia) over the 12-month follow up period after the third vaccine dose.
